# A dual-therapy approach for the treatment of biofilm-mediated *Salmonella* gallbladder carriage

**DOI:** 10.1371/journal.ppat.1009192

**Published:** 2020-12-28

**Authors:** Jenna L. Sandala, Bradley W. Eichar, Laura G. Kuo, Mark M. Hahn, Akash K. Basak, William M. Huggins, Katherine Woolard, Christian Melander, John S. Gunn

**Affiliations:** 1 Center for Microbial Pathogenesis, Abigail Wexner Research Institute at Nationwide Children’s Hospital, Columbus, Ohio, United States of America; 2 Department of Microbial Infection and Immunity, The Ohio State University College of Medicine, Columbus, Ohio, United States of America; 3 Infectious Diseases Institute, The Ohio State University, Columbus, Ohio, United States of America; 4 Department of Chemistry and Biochemistry, University of Notre Dame, Notre Dame, Indiana, United States of America; 5 Department of Chemistry, North Carolina State University, Raleigh, North Carolina, United States of America; 6 Department of Pediatrics, The Ohio State University College of Medicine, Columbus, Ohio, United States of America; University of California Davis School of Medicine, UNITED STATES

## Abstract

Asymptomatic carriage of *Salmonella* Typhi continues to facilitate the transmission of typhoid fever, resulting in 14 million new infections and 136,000 fatalities each year. Asymptomatic chronic carriage of *S*. Typhi is facilitated by the formation of biofilms on gallstones that protect the bacteria from environmental insults and immune system clearance. Here, we identified two unique small molecules capable of both inhibiting *Salmonella* biofilm growth and disrupting pre-formed biofilm structures without affecting bacterial viability. In a mouse model of chronic gallbladder *Salmonella* carriage, treatment with either compound reduced bacterial burden in the gallbladder by 1–2 logs resulting in bacterial dissemination to peripheral organs that was associated with increased mortality. Co-administration of either compound with ciprofloxacin not only enhanced compound efficacy in the gallbladder by a further 1–1.5 logs for a total of 3–4.5 log reduction, but also prevented bacterial dissemination to peripheral organs. These data suggest a dual-therapy approach targeting both biofilm and planktonic populations can be further developed as a safe and efficient treatment of biofilm-mediated chronic *S*. Typhi infections.

## Author summary

Typhoid fever is an infectious disease caused by *Salmonella* Typhi (*S*. Typhi), a bacterium that causes as many as 14 million new infections and 136,000 deaths annually. Asymptomatic chronic carriers of *S*. Typhi play a major role in the transmission of typhoid fever, as they intermittently shed the bacteria and can unknowingly infect surrounding individuals. Here, we characterized novel compounds that target biofilm formation, a process utilized by *S*. Typhi to establish and maintain chronic carriage in the gallbladder, in hopes that they may be eventually used in conjunction with traditional antibiotics to prevent and/or cure chronic infections more efficiently than antibiotics alone.

## Introduction

*Salmonella enterica* serovar Typhi (*S*. Typhi) is the etiological agent of typhoid fever, an acute systemic infection that is characterized by fever, malaise, and abdominal pain. *S*. Typhi is a human-restricted pathogen and is transmitted via the fecal-oral route [1–3]. Typhoid fever is therefore rarely reported in more developed countries such as the United States; however, the disease remains endemic in a number of developing regions where sanitation is relatively poor, resulting in 14 million new infections and 136,000 deaths each year [4]. While the use of fluoroquinolone antibiotics such as ciprofloxacin resolve the majority of acute typhoid infections, it is estimated that 3–5% of acutely infected individuals will develop a chronic infection and continue to harbor *S*. Typhi after symptoms have subsided [3,5,6]. Such asymptomatic chronic carriage presents a major public health problem in endemic regions, as carriers unknowingly serve as a reservoir for typhoidal serovars and remain capable of spreading the disease via fecal contamination [3]. Chronic *S*. Typhi infections are primarily localized to the gallbladder and are facilitated by the formation of biofilms on cholesterol gallstones [3,6–9].

Bacterial biofilms are highly structured communities of bacteria encased within a matrix of extracellular polymeric substances. Entry into the biofilm phenotype is a sequential process during which planktonic (free-floating) bacteria attach to a surface, grow, and assemble an extracellular matrix from self-secreted and/or environmentally acquired polymeric substances including carbohydrates, proteins, lipids, and extracellular DNA [10,11]. Once the biofilm has matured, individual bacteria or biofilm aggregates can disperse from the main structure and colonize new sites. In the context of human infection, biofilm formation provides bacteria with a fitness advantage relative to the planktonic phenotype by conferring increased resistance to harsh environmental conditions (e.g. gallbladder bile), host immune effectors, and antibiotic therapies [12–16]. In line with these observations, traditional antibiotic therapy only shows moderate success against chronic typhoid infections. Finally, while cholecystectomy has been shown to eliminate carriage, it is costly, invasive, and thus impractical to be widely adopted in affected regions [3,5,17–21].

**Fig 1:**
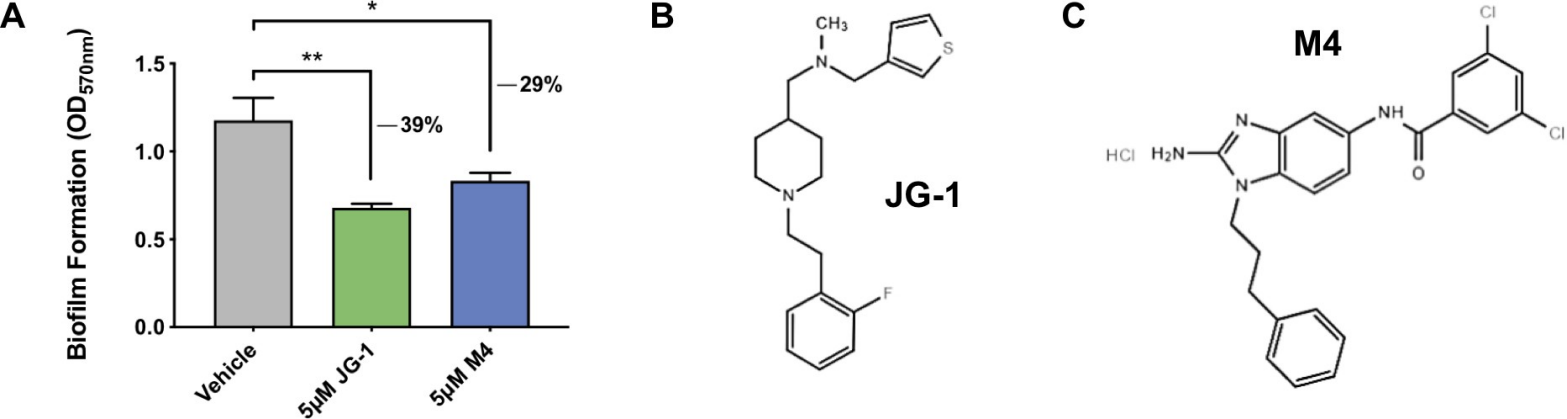
Identification of small molecule inhibitors of *Salmonella* Typhimurium biofilm formation. **A.** The compounds JG-1 and M4 were identified as inhibitors of *Salmonella* Typhimurium (*S*. Typhimurium) biofilm formation via high-throughput screening of small molecule libraries. *S*. Typhimurium biofilms were grown in microtiter plates in the presence of various compounds at a concentration 5 μM. Biofilm formation after 24 h was evaluated in a semi-quantitative manner using the crystal violet assay, and inhibition of biofilm formation was calculated by assessing the amount of biofilm formed relative to a vehicle control (DMSO). Data are presented as mean +/- SD; ** p < 0.01, * p < 0.05, as assessed by unpaired t-tests. **B and C.** Chemical structures of the small molecule biofilm inhibitors JG-1 **(B)** and M4 **(C)**.

Because of the importance of biofilms in chronic carriage, the use of anti-biofilm agents is a promising strategy to eliminate gallbladder carriage and subsequently reduce the spread of typhoid fever. Small molecules that target bacterial factors involved in biofilm formation and maintenance have been successfully used to inhibit and/or disperse biofilms produced by a number of bacterial pathogens [22–30]. Using a high-throughput screening approach, we identified two unique small molecules–JG-1 and M4 –that inhibit *Salmonella* biofilms *in vitro* (Fig 1). Here, we further characterized the anti-biofilm capabilities of these compounds *in vitro* and assessed their therapeutic potential using a mouse model of chronic *Salmonella* Typhimurium (*S*. Typhimurium) gallbladder carriage.

## Results

### Inhibition of *Salmonella* biofilm formation by the small molecules JG-1 and M4

We sought to identify compounds with anti-biofilm activity against *Salmonella* by performing two separate small molecule library screens, which led us to identify the compounds JG-1 and M4 (Fig 1). The 2-aminobenzamidazole M4 was discovered in a screen published previously [23]. The compound JG-1 was initially identified by screening a library of 4,000 small molecules each at 5 μM purchased from ChemBridge (San Diego, CA). We then identified any compounds capable of inhibiting *S*. Typhimurium biofilm formation by at least 30% relative to a vehicle control. After screening initial hits of the ChemBridge library for maintained anti-biofilm activity, JG-1 was the only compound that (in addition to M4) warranted further characterization.

**Fig 2:**
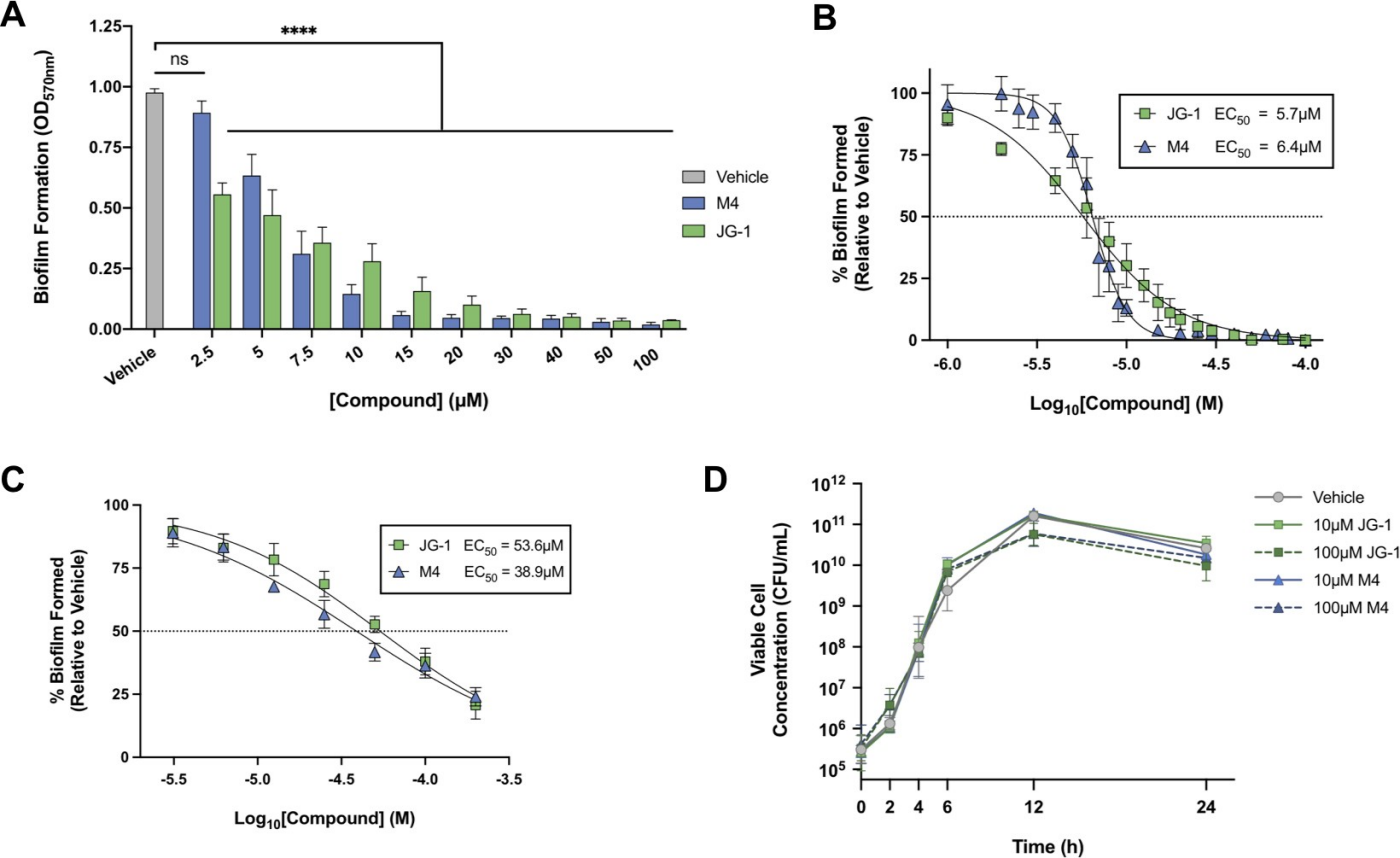
JG-1 and M4 inhibit *Salmonella* biofilm formation in a dose-dependent manner without affecting planktonic growth. **A.** *S*. Typhimurium biofilms were grown for 24 h in the presence of a vehicle control (DMSO) or various concentrations (2.5–100 μM) of JG-1 or M4 in order to assess the dose-dependency of biofilm inhibition. Biofilm formation was measured using the crystal violet assay. Annotations above the data denote values that are significantly different from the vehicle control (gray bar), as determined by two-way ANOVA with Dunnett’s test for multiple comparisons; ns p ≥ 0.05, **** p < 0.0001. **B.** *S*. Typhimurium biofilms were grown as described in **A**, and EC_50_ values for JG-1 [5.7μM (95% CI, 5.2–6.2 μM)] and M4 [6.4μM (95% CI, 6.3–6.6 μM)] were calculated using GraphPad Prism 8 to plot normalized compound activity (percent biofilm formed) as a function of log_10_ drug concentration and fitting a dose-response curve (log[inhibitor] vs. normalized response, variable slope). **C.** *S*. Typhi biofilms were grown in cholesterol-coated microtiter plates for a total of 96h in the presence of a vehicle control (DMSO) or various concentrations (3.1–200 μM) of JG-1 or M4. Corresponding EC_50_ values for JG-1 [53.6 μM (95% CI, 46.4–62.3 μM)] and M4 [38.9μM (95% CI, 33.6–45.2μM)] were calculated as described in **B**. **D.** JG-1 and M4 have no effect on the viability of planktonic *S*. Typhimurium. Liquid *S*. Typhimurium cultures were grown in the presence of JG-1, M4, or a vehicle control (DMSO) for a total of 24 h. At various timepoints, aliquots of cultures were serially diluted and plated onto LB for CFU enumeration. Two-way ANOVA with the Tukey correction for multiple comparisons revealed no significant differences at any timepoint. All data are presented as the mean +/- SD.

*S*. Typhi is a human-specific serovar and is unable to colonize mice; however, the related serovar *S*. Typhimurium (utilized in the screen) is often used to model typhoid infection in mice, as it recapitulates many characteristics of the human disease. Therefore, we assessed the anti-biofilm capabilities of JG-1 and M4 using both *S*. Typhimurium and *S*. Typhi. To directly compare abilities of JG-1 and M4 to inhibit *Salmonella* biofilm formation, biofilms grown in media containing either a vehicle control (DMSO) or varying concentrations of compound were stained with crystal violet (Fig 2A). While both JG-1 and M4 exhibited a dose-response relationship and were similarly capable of inhibiting biofilm formation in *S*. Typhimurium (Fig 2B; EC_50_ = 5.7 μM and 6.4 μM, respectively), M4 inhibited *S*. Typhi biofilms more efficiently than did JG-1 (Fig 2C; EC_50_ = 38.9 μM and 53.6 μM, respectively). In order to determine if the anti-biofilm effects of JG-1 and M4 could be attributed to bacteriostatic or bactericidal activities, liquid cultures of *S*. Typhimurium were incubated in the presence of vehicle or a low (10 μM) or high (100 μM) concentration of either compound for a total of 24 h. No significant differences in growth progression were observed at any of the timepoints assessed (Fig 2D).

### JG-1 and M4 disrupt pre-formed *Salmonella* biofilms

**Fig 3:**
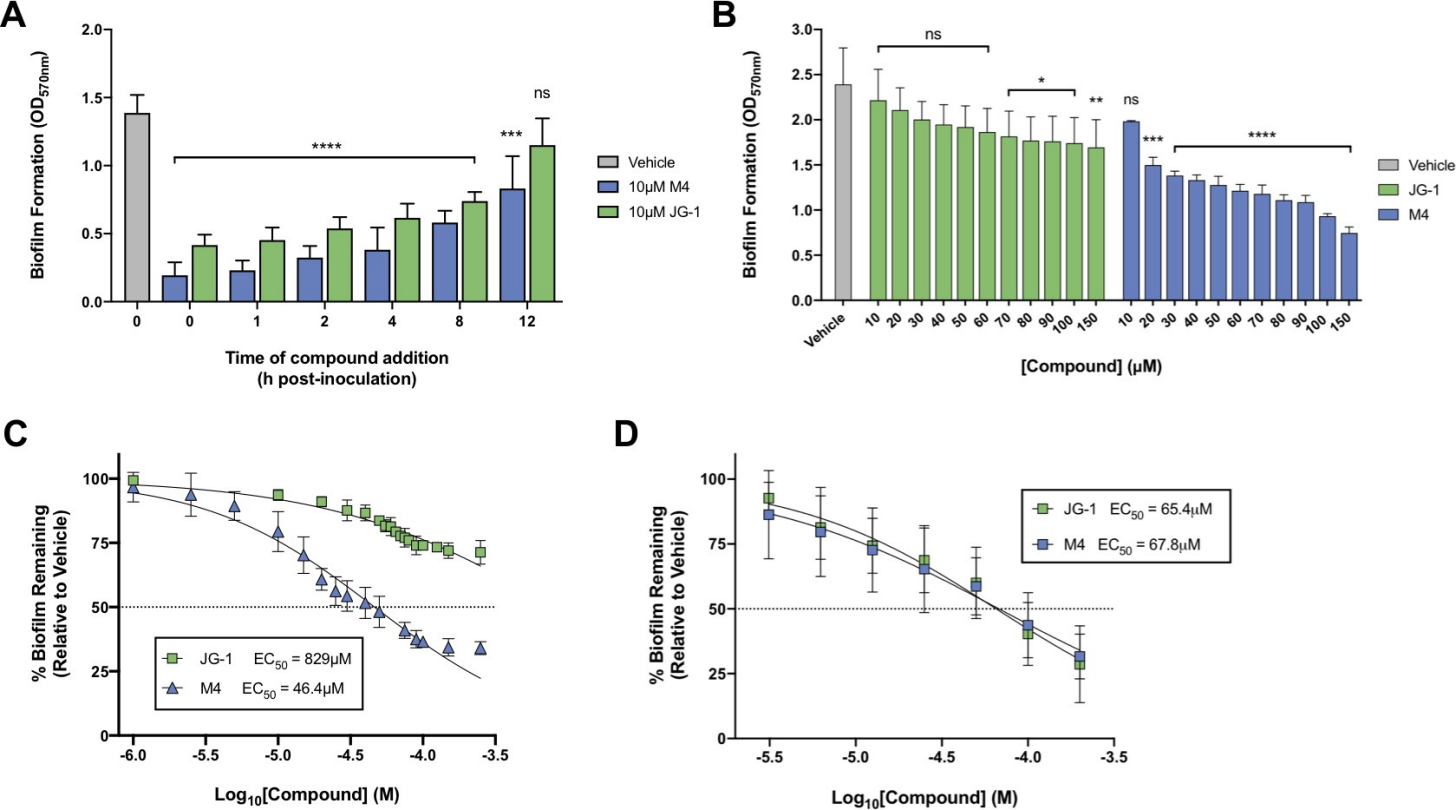
Disruption of preformed *Salmonella* biofilms by JG-1 and M4. **A.** Addition of JG-1 or M4 to developing *S*. Typhimurium biofilms suggests anti-biofilm activity decreases as biofilm development progresses. At either the time of inoculation (0h post-inoculation) or at various timepoints 1–12 h post-inoculation, JG-1 or M4 was added to the media of *S*. Typhimurium biofilms to achieve a final compound concentration of 10 μM. Biofilms were incubated for a total of 24 h, after which biofilm formation relative to a vehicle control (DMSO) was assessed via crystal violet assay. Annotations above the data denote values that are significantly different from the vehicle control (gray bar), as determined by two-way ANOVA with Dunnett’s test for multiple comparisons; ns p ≥ 0.05, *** p < 0.001, **** p < 0.0001. **B.** Untreated *S*. Typhimurium biofilms were grown for 24h, after which media was removed and replaced with various concentrations (10–150 μM) JG-1 or M4 or a vehicle control (DMSO). Incubation was resumed for an additional 24 h, after which biofilm formation was assessed via crystal violet assay. Annotations above the data denote values that are significantly different from the vehicle control (gray bar), as determined by two-way ANOVA with Dunnett’s test for multiple comparisons; ns p ≥ 0.05, * p < 0.05, ** p < 0.01, *** p < 0.001, **** p < 0.0001. **C.** *S*. Typhimurium biofilms were grown and treated as described in **B** and EC_50_ values for biofilm disruption with JG-1 [829.4 μM (95% CI, 608.1 μM–1.2 mM)] and M4 [46.4 μM (95% CI, 41.6–52.1 μM)] were calculated using GraphPad Prism 8 to plot normalized compound activity (percent biofilm remaining) as a function of log_10_ drug concentration and fitting the dose response curve (log[inhibitor] vs. normalized response, variable slope). **D.** *S*. Typhi biofilms were grown in cholesterol-coated microtiter plates for 96h, then treated with a vehicle control (DMSO) or various concentrations (3.1–200 μM) JG-1 or M4 for 24 h. Corresponding EC_50_ values for biofilm disruption were calculated for JG-1 [67.8μM (95% CI, 43.0–124.6 μM)] and M4 [65.4 (95% CI, 46.5–97.6 μM)] as described in **C**. All data are presented as the mean +/- SD.

While inhibiting biofilm formation can be useful in certain situations, disruption of pre-existing biofilm structures is of greater therapeutic relevance and value due to the current absence of biofilm disruption therapies or highly successful treatments for *Salmonella* chronic carriers. We assessed the relative anti-biofilm effects of JG-1 and M4 at various stages during biofilm development by exposing *S*. Typhimurium biofilms to either compound at various timepoints 1–12 h post-inoculation. While delaying the addition of 10 μM JG-1 or M4 to developing *S*. Typhimurium biofilms was correlated with a reduction in anti-biofilm effects, both compounds were still able to significantly inhibit biofilm maturation when added as late as 8 h post-inoculation (Fig 3A).

We next investigated whether JG-1 or M4 could disrupt mature biofilms by assessing the effects of treating 24 h biofilms with either compound at various concentrations (3.1–200 μM). Both JG-1 and M4 partially disrupted pre-formed *Salmonella* biofilms in a dose-dependent manner (Fig 3B); however, M4 was capable of disrupting *S*. Typhimurium biofilms to a greater extent than JG-1 (Fig 3C; EC_50_ = 46.4 μM and 829 μM, respectively). In contrast, both compounds were similarly capable of disrupting *S*. Typhi biofilms (Fig 3D; JG-1 EC_50_ = 65.4 μM, M4 EC_50_ = 67.8 μM).

### Concurrent administration with ciprofloxacin augments the biofilm disrupting capabilities of JG-1 and M4

**Fig 4:**
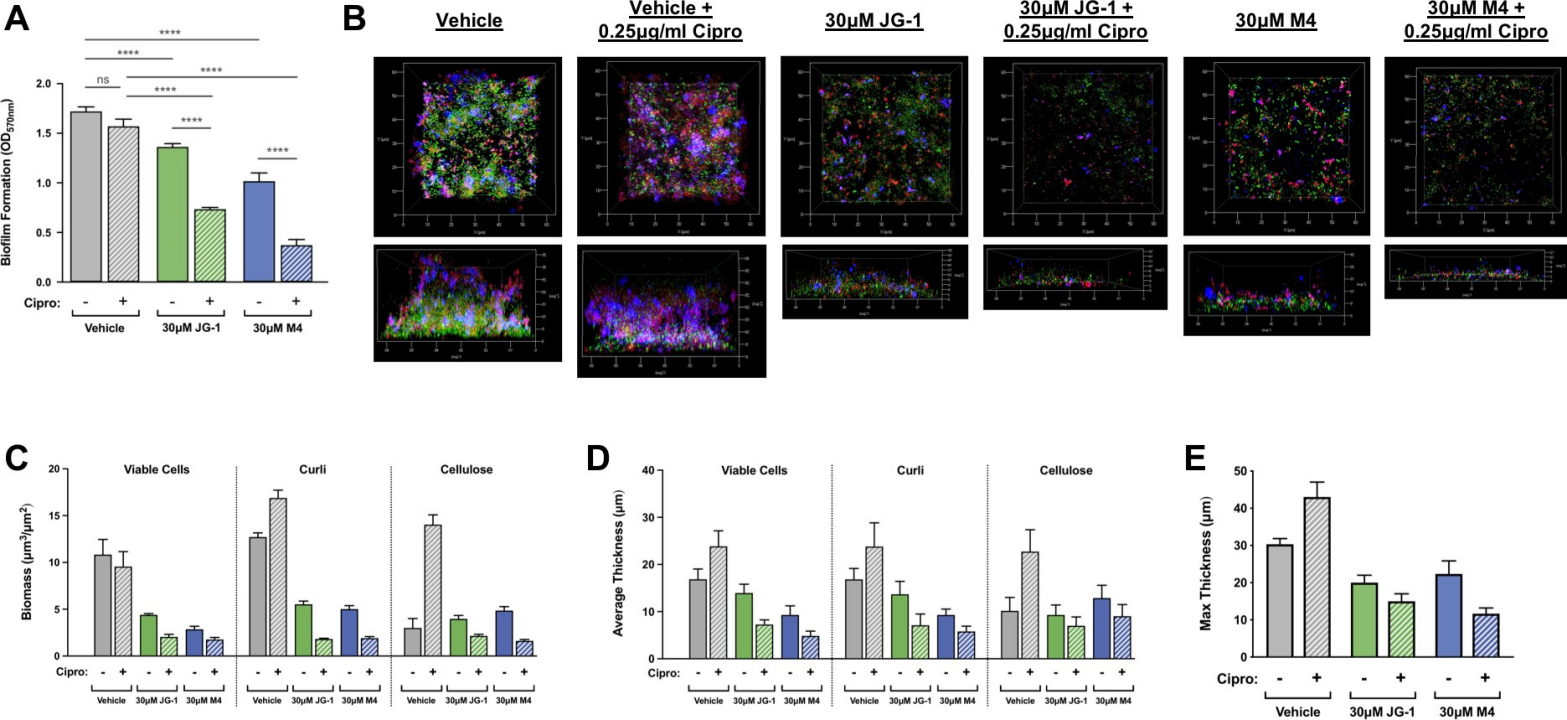
Concurrent treatment with ciprofloxacin (cipro) enhances the anti-biofilm capabilities of JG-1 and M4. **A.** *S*. Typhimurium biofilms were grown in microtiter plates for 24 h, then treated for 24h with a vehicle control (DMSO), 0.25 μg/mL cipro, 30 μM JG-1/M4, or a combination of 0.25 μg/mL cipro + 30 μM JG-1/M4. Biofilm formation was measured using the crystal violet assay and graphed as the mean +/- SD. **B.** *S*. Typhimurium biofilms were grown in 8-well glass-bottom chamber slides for 96 h, then treated for 24h with a vehicle control (DMSO), 0.25μg/mL cipro, 30 μM JG-1/M4, or a combination of 0.25 μg/mL cipro + 30 μM JG-1/M4. Biofilms were stained with a combination of Syto9 and Calcofluor white in order to visualize viable cells (green) and cellulose (blue), respectively, prior to fixation in 2% PFA. Curli fimbriae (red) were visualized using a human α-amyloid mAb. Representative z-stacks (N = 5 per treatment) were captured at 100X magnification using a Zeiss LSM 800 laser scanning confocal microscope. **C–E.** COMSTAT2 software was used to calculate the biomass **(C)**, average thickness **(D)**, and maximum thickness **(E)** of biofilm z-stacks for each individual component (cells, cellulose, and curli). Data are presented as the mean +/- SD.

We previously demonstrated that compared to their planktonic counterparts, *Salmonella* within a biofilm exhibit increased sensitivity to ciprofloxacin both *in vitro* and *in vivo* in a mouse model of chronic carriage [31]. As ciprofloxacin is a first-line therapy for both acute and chronic typhoidal infections in most countries, we utilized it as a benchmark agent to further assess the therapeutic potential of JG-1 and M4 against *Salmonella* biofilms. In line with our previous data, treatment of 24 h *S*. Typhimurium biofilms (testing dispersal) with 0.25 μg/mL ciprofloxacin (the MIC for planktonic cells) had no apparent effect, while treatment with 30 μM JG-1 or M4 both resulted in a significant reduction in biofilm as determined by crystal violet staining (Fig 4A). Interestingly, we also explored the effects of combining either JG-1 or M4 with ciprofloxacin and found that the addition of ciprofloxacin greatly enhanced the disruptive effects of both compounds despite ciprofloxacin exhibiting no anti-biofilm effects on its own (Fig 4A).

In order to visualize and compare the effects of anti-biofilm treatments on *Salmonella* biofilm structure, we used confocal microscopy to image *S*. Typhimurium biofilms that were grown in glass-bottom chamber slides for 96h prior to treatment. To differentiate between bacterial cells and the extracellular matrix, biofilms were stained for viable cells, cellulose, and curli fimbriae prior to imaging and subsequent structural analysis with COMSTAT2 software [32]. While ciprofloxacin treatment did result in a slight loss of viable cells compared to the vehicle control, it actually caused an increase in the biomass of both cellulose and curli, as well as an increase in both the average and maximum thickness of the biofilm (Fig 4B–4E). In contrast, treatment with JG-1 or M4 alone decreased the biomass of viable cells, cellulose, and curli to a similar extent (Fig 4B and 4C), resulting in biofilms that were visibly thinner and patchier than the thick and confluent untreated controls (Fig 4B, 4D and 4E). Combining either JG-1 or M4 with ciprofloxacin magnified these effects such that the biofilm was reduced to a collection of thin microcolonies that sparsely adhered to the chamberglass surface (Fig 4B–4E).

### Combination with ciprofloxacin enhances the safety and efficacy of JG-1 and M4 treatments in a mouse model of chronic *Salmonella* gallbladder carriage

**Fig 5:**
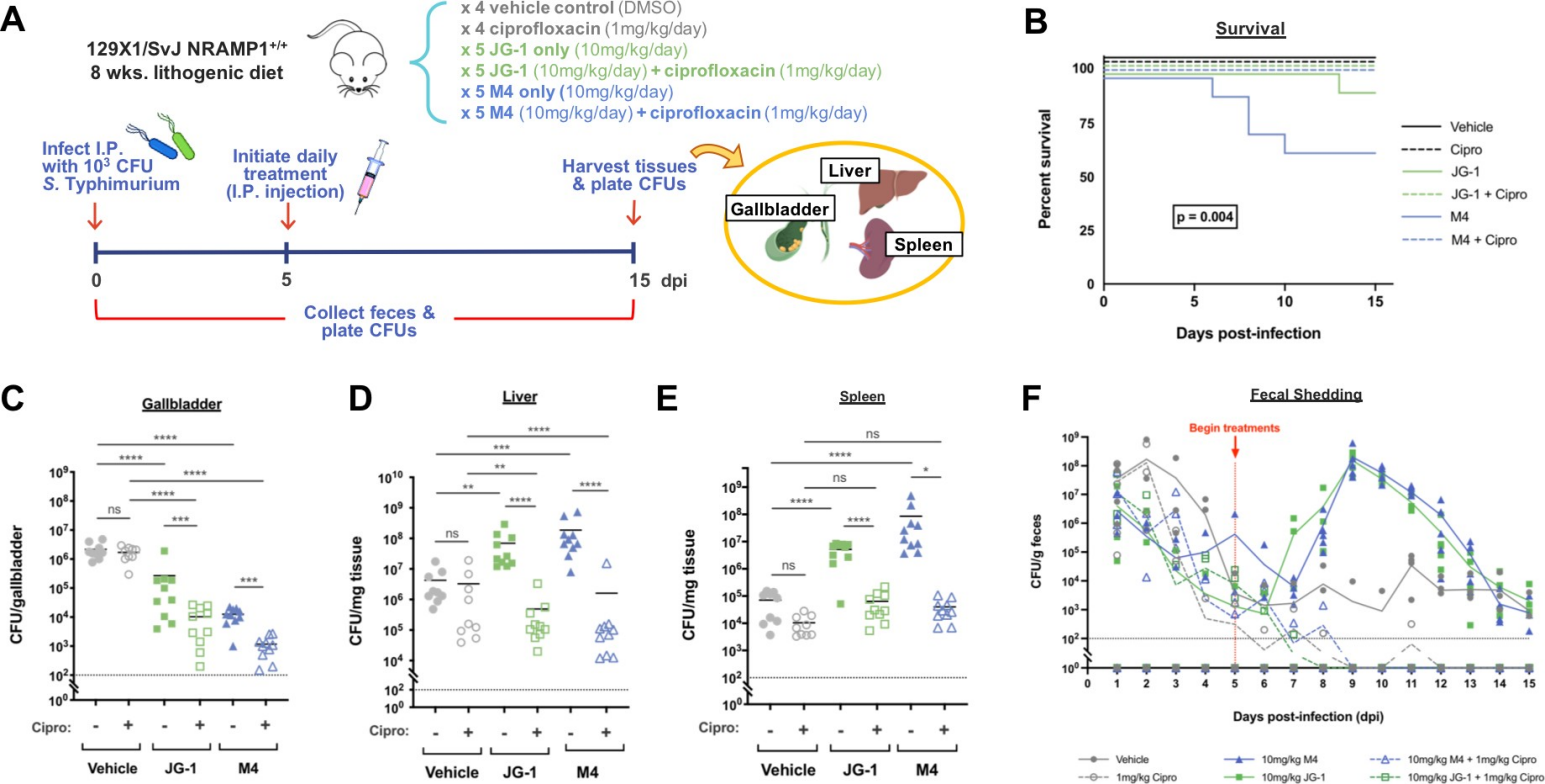
Combination with ciprofloxacin (cipro) enhances the safety and efficacy of JG-1 and M4 in a mouse model of chronic gallbladder carriage of *Salmonella*. **A.** Experimental design: 129X1/SvJ mice were fed a lithogenic diet for 8 wks. prior to I.P. infection with 10^3^ *S*. Typhimurium. Starting 5 days post-infection (dpi) and continuing through 15 dpi, mice received daily I.P. treatments of cipro, JG-1, M4, JG-1 + cipro, M4 + cipro, or a vehicle control. CFU enumeration was used as a means of evaluating bacterial burden in various organs at 15 dpi as well as fecal shedding of *Salmonella* throughout the course of infection. **B.** Survival analysis of *Salmonella-*infected mice receiving various treatments. Kaplan Meier survival estimates were evaluated for overall variance using the logrank test. Post hoc pairwise comparisons of M4 and JG-1 curves were performed using the logrank test with the Bonferroni correction for multiple comparisons (adjusted threshold for significance: p < 0.025). **C–E.** Enumeration of *Salmonella* in the gallbladder **(C)**, liver **(D)**, and spleens **(E)** of infected mice on 15 dpi. ns non-significant, * p < 0.05, ** p < 0.01, *** p < 0.001, **** p < 0.0001. **F.** Fecal burden of *Salmonella* in infected mice monitored from 0 (prior to infection) to 15 (prior to sacrifice) dpi. Images created with BioRender.com.

After observing JG-1 and M4 consistently disrupt pre-formed *Salmonella* biofilms *in vitro*, we devised experiments to address whether their activity would be maintained in an *in vivo* model. Initial toxicity assays performed using HeLa cells and *Galleria mellonella* moth larvae suggested that neither compound is toxic at concentrations sufficient for *in vitro* anti-biofilm activity, and dose escalation studies in C57BL/6 mice suggested that intraperitoneal (I.P.) administration of either compound was well tolerated at doses up to and including the max feasible dose (due to solubility) of 10 mg/kg/day over a period of 10 days (no observed adverse effects, 0/5 mice for each compound). Given these results, we decided to further explore the therapeutic potential of JG-1 and M4, alone and in combination with ciprofloxacin, using our established mouse model of chronic *Salmonella* gallbladder carriage [6]. In this model, 129x1/SvJ NRAMP^+/+^ mice are fed a lithogenic diet (standard chow supplemented with 1% cholesterol and 0.5% cholic acid) for eight weeks in order to induce the formation of cholesterol gallstones. Subsequent infection with *S*. Typhimurium results in biofilm-mediated carriage in the gallbladder, similar to what is observed with *S*. Typhi in humans. In the present study, mice were randomized to one of six 10-day I.P. treatment regimens: ciprofloxacin alone (1 mg/kg/day), JG-1 or M4 alone (10 mg/kg/day), a combination of ciprofloxacin (1 mg/kg/day) and either JG-1 or M4 (10 mg/kg/day), or a vehicle control (DMSO). In order to allow sufficient time for *S*. Typhimurium to reach the gallbladder and form a biofilm, treatment regimens were initiated five days post-infection (dpi) and continued through 15 dpi. Mice were then sacrificed and their gallbladders, livers, and spleens were harvested for assessment of *Salmonella* burden (Fig 5A).

In agreement with previous reports, ciprofloxacin alone was incapable of reducing *Salmonella* burden in the gallbladder of infected mice (Fig 5C), though it did result in a slight but insignificant reduction in bacterial burden in the liver and the spleen (Fig 5D and 5E). In contrast, treatment with JG-1 or M4 alone reduced bacterial burden in the gallbladder by 1–2 logs respectively (Fig 5C), but surprisingly caused an increase in the number of bacteria recovered from the liver and spleen (Fig 5D and 5E). These animals also experienced greater mortality, which is atypical for this particular model of infection (Fig 5B). To determine if treatment with JG-1 and M4 was perhaps causing a more invasive phenotype that resulted in not only increased dissemination to the liver and spleen but also increased invasion of the gallbladder, we separated the gallbladder tissue from the gallbladder contents (gallstones and bile) prior to enumeration. This revealed that the reduction in total gallbladder burden following treatment with JG-1 or M4 was primarily due to a reduction in the gallbladder contents (1–2.2 logs), but that there was also a slight reduction in bacteria associated with the gallbladder tissue (<1 log; S1 Fig).

Feces were also collected daily throughout the infection period in order to monitor the effect of treatments on fecal shedding patterns (Fig 5F). The shedding patterns mirrored the dissemination data, with increased fecal bacteria in mice treated with the compounds alone versus in combination with ciprofloxacin. As observed with *in vitro* biofilms, co-administration of ciprofloxacin with JG-1 or M4 further reduced *Salmonella* gallbladder burden by another 1–2 logs vs. JG-1 or M4 alone (Fig 5C), and the bacterial burden in the liver and spleen 2–3 logs vs. JG-1 or M4 alone (Fig 5D and 5E). The bacterial burden in the liver and spleen with ciprofloxacin plus M4 or JG-1 approximated the vehicle control group, however, there was no associated mortality (Fig 5B). Thus, the biofilm dispersal compounds M4 and JG-1 effectively, safely and significantly reduced chronic carriage in the mouse model.

## Discussion

*S*. Typhi, the etiological agent of typhoid fever, continues to cause significant morbidity and mortality in affected regions [1–4]. While the most severe consequences of acute *S*. Typhi infection can typically be avoided with early diagnosis and appropriate antibiotic intervention, the high costs associated with treatment–as well as concerns of emerging antibiotic resistance–continue to drive efforts to reduce disease transmission [3,4]. As *S*. Typhi is a human-specific pathogen with no known vectors or environmental reservoirs, chronic carriers play an essential role in the cycle of *S*. Typhi infection and transmission by serving as long-term hosts and facilitating disease transmission via intermittent shedding of viable bacteria in their feces [3,6–9]. Transition to the biofilm state permits chronic carriage in the gallbladder and makes *S*. Typhi tolerant to host immune effectors and antibiotics [3,5,12–21]. Thus, therapeutics that effectively interfere with the *S*. Typhi biofilm state could help to resolve chronic infection and consequently reduce disease transmission.

Toward this end, we used a high-throughput approach to screen over 4,000 small molecules for the ability to inhibit *Salmonella* biofilm formation and identified JG-1 (Fig 1). JG-1 and the previously identified M4 both inhibited *in vitro* biofilm formation in a dose-dependent manner for both *S*. Typhi and *S*. Typhimurium, a related serovar used to model typhoidal disease in mice (Fig 2B and 2C**)**. Importantly, the anti-biofilm effects of both JG-1 and M4 appear to be specific to the biofilm phenotype, as *S*. Typhimurium cultured in liquid media containing either compound exhibited no significant growth defect compared to a vehicle control (Fig 2D). This specificity makes it unlikely that their use would select for resistant mutants in planktonic populations.

While inhibiting *Salmonella* biofilm formation may be useful in certain situations (for example, as a means of preventing chronic colonization of the gallbladder in acutely infected individuals), the ability to disrupt pre-existing biofilm structures is of greater clinical relevance and value, as there are currently no therapies that reliably resolve chronic carriage [17–21]. We therefore sought to determine whether JG-1 and M4 would be capable of disrupting developing and/or mature (pre-formed) *Salmonella* biofilms by delaying the addition of compounds to *in vitro* biofilms. While the anti-biofilm activities of both compounds did gradually decrease over 24 h of *S*. Typhimurium biofilm development, both JG-1 and M4 were capable of partially disrupting mature *S*. Typhimurium and *S*. Typhi biofilms (Fig 3).

Experimental induction of biofilm disruption or dispersal is typically associated with a phenotypic switch to a planktonic-like state [33]. This phenotype is often characterized by a reduction in antimicrobial tolerance with released bacteria exhibiting levels of antimicrobial susceptibility that are similar to those of their planktonic counterparts [34–44]. In such cases, the combination of anti-biofilm compounds with traditional antibiotics results in an even greater amount of biofilm clearance. We therefore sought to determine if combining either JG-1 or M4 with ciprofloxacin, one of the most common first-line treatments for *S*. Typhi infections, would result in even greater levels of biofilm disruption compared to either compound alone. Indeed, while treatment of *S*. Typhimurium biofilms with ciprofloxacin alone had minimal anti-biofilm effects as determined by both crystal violet staining and confocal microscopy, combination treatments consisting of ciprofloxacin and either JG-1 or M4 resulted in a significantly greater reduction in biofilm than did treatment with the respective anti-biofilm compound alone (Fig 4), suggesting that disruption of *Salmonella* biofilms by JG-1 or M4 is associated with a decrease in the antimicrobial tolerance associated with the biofilm state.

Observing the promising anti-biofilm capabilities of JG-1 and M4 against *Salmonella in vitro* led us to question whether these compounds could be used therapeutically *in vivo*. To address this question, we utilized an established mouse model of *Salmonella* biofilm-mediated gallbladder carriage to assess the therapeutic effects of various treatment regimens including a vehicle control (DMSO), ciprofloxacin alone, JG-1 or M4 alone, and JG-1 or M4 in combination with ciprofloxacin (Fig 5A). For treatments including ciprofloxacin, a concentration of 1 mg/kg/day ciprofloxacin was chosen as we have previously shown this concentration significantly reduces gallbladder burden in a model of acute infection but not in a model of chronic infection [31]. JG-1 and M4 were supplied at a concentration of 10 mg/kg/day, as this was the maximum feasible dose for either compound due to solubility. Additionally, administration of JG-1 or M4 at this concentration was confirmed to be well-tolerated by uninfected mice. In concordance with our previous results, treatment with ciprofloxacin alone was unable to significantly reduce bacterial burden in the gallbladder of infected mice, likely due to the formation of biofilms on gallstone surfaces (Fig 5C). However, ciprofloxacin alone did result in a slight but statistically insignificant reduction in bacterial burden in the liver and spleen, as well as in the feces, where any *Salmonella* are likely not biofilm-associated and thus susceptible to the mechanisms of traditional antibiotics (Fig 5D–5F). In contrast, treatment with JG-1 or M4 alone resulted in a significant reduction in bacterial burden in the gallbladder (Fig 5C), but also unexpectedly caused an increase in bacterial burden in the liver, spleen, and feces (Fig 5D–5F), as well as atypical levels of mortality (Fig 5B). Combination treatment with either JG-1 or M4 and ciprofloxacin not only further reduced the number of bacteria in the gallbladder compared to each respective treatment alone (Fig 5C), but also prevented the unexpected peripheral accumulation of bacteria and associated mortality observed with JG-1 and M4 treatment alone (Fig 5B–5F). Since ciprofloxacin is likely having an effect only on planktonic/released bacteria, increasing the antibiotic dose would further improve clearance from peripheral organs as there is no evidence to suggest biofilms form at these sites. Additionally, we demonstrated that the majority of released bacteria come from the gallbladder contents (i.e. gallstones) and not the gallbladder tissue (S1 Fig). It is possible that the reduction of CFU observed in the gallbladder tissue post M4/JG-1 treatment was also due to biofilm dispersal, as we previously observed biofilm formation on the luminal side of the epithelium in our mouse model.

**Fig 6:**
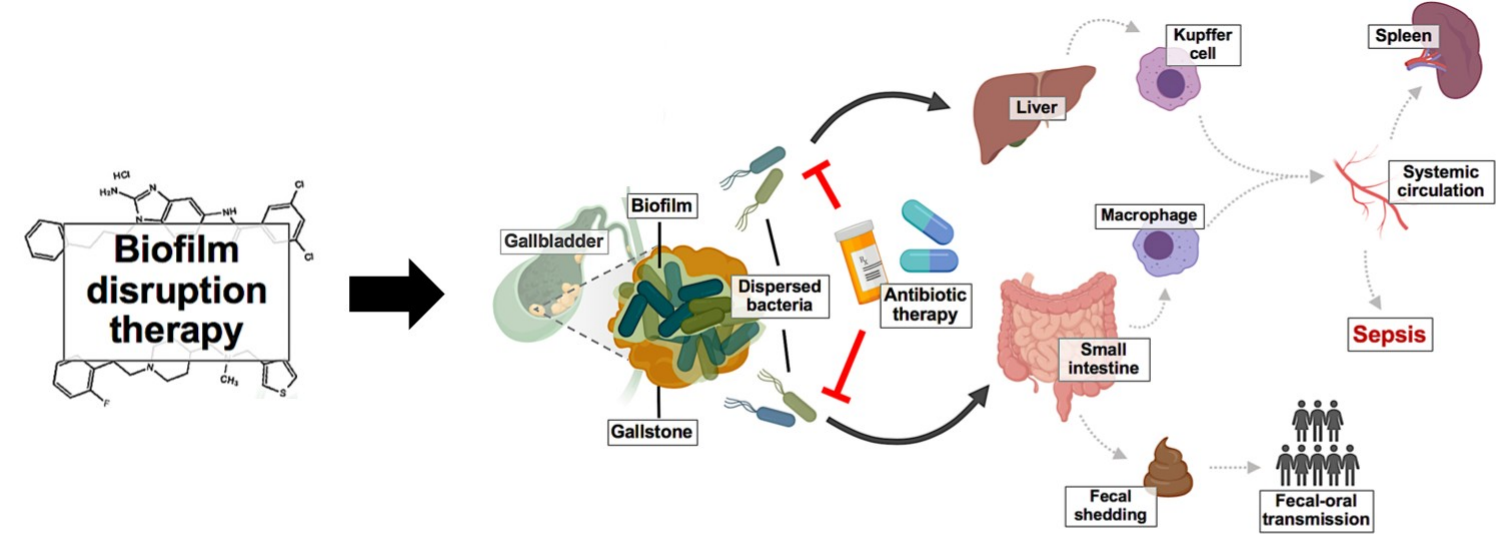
Model of a dual therapy approach for the safe and efficient disruption of *Salmonella* biofilms *in vivo*. Following treatment with anti-biofilm agents, *Salmonella* biofilms on the surface of gallstones are disrupted, and the released bacteria disseminate via the biliary tract to the small intestine and/or liver. Here, disseminated *Salmonella* can proliferate and cause tissue damage, or escape phagocytosis and gain access to the bloodstream resulting in systemic infection, sepsis, or even death. Dissemination of *Salmonella* to the small intestine can also lead to fecal contamination and increasing the risk of transmission. Concurrently administering traditional antibiotic therapy with anti-biofilm agents mitigates the aforementioned risks by killing bacteria as they are released from the biofilm, and thereby serves as a safe and efficient means of treating biofilm-mediated chronic carriage of *Salmonella*. Images created with BioRender.com.

Why might treatment with anti-biofilm compounds alone increase bacterial burden in peripheral organs as well as an increase in mortality? We hypothesize that because JG-1 and M4 disrupt biofilm cohesiveness without affecting viability of biofilm-associated *Salmonella*, the bacteria released following treatment with either of these compounds alone are able to descend to the intestine (or ascend to the liver via the biliary tract). There they invade the epithelium and are subsequently engulfed by macrophages (or Kupffer cells) and subsequently disseminate throughout the body to colonize peripheral organs, potentially resulting in increased fecal shedding, organ dysfunction, septic shock, or even death. Similar reports of bacteria dissemination and colonization of secondary sites following experimentally-induced biofilm disruption have previously been published for other organisms, including *Pseudomonas aeruginosa* and *Candida albicans* [37,45]. Furthermore, recent studies utilizing RNAseq have revealed that bacterial cells released following biofilm disruption have a unique transcriptional profile that often results in a more virulent phenotype compared to biofilm-associated or broth-grown planktonic cells [34,35,40,46–50]. Therefore, we predict *Salmonella* has a similar virulence phenotype occurring following the disruption of biofilms, which would explain the mortality associated with JG-1 and M4 treatments. Fortunately, combining either JG-1 or M4 with ciprofloxacin not only prevented the dissemination of released bacteria to other organs, but also resulted in enhanced bacterial clearance at all locations examined. While ciprofloxacin resistance is relatively low worldwide [51], the use of alternative antibiotics (to mitigate the risk of antimicrobial resistance) such as ceftriaxone or azithromycin could likely achieve a similar effect as the synergistic activity of combining anti-biofilm agents with antibiotics has been reported for multiple classes of antibiotics [34–44]. Thus, we propose that in order to safely and efficiently clear biofilm-mediated chronic gallbladder carriage of *Salmonella*, a dual-therapy approach targeting both biofilm and planktonic populations should be pursued (Fig 6).

In summary, we have identified two small molecules that are capable of both inhibiting and disrupting *Salmonella* biofilms *in vitro* without affecting bacterial viability. Both compounds exhibit increased disruptive capabilities when administered in combination with ciprofloxacin, suggesting that they act synergistically with traditional antibiotics. To our knowledge, ours is the first study to assess the therapeutic potential of anti-biofilm agents in an animal model of *Salmonella* carriage. Further, these results confirm that the biofilm lifestyle is key to persistence in the gallstone-containing gallbladder. Our results show that while treatment of chronic carriage with anti-biofilm compounds alone may be able to reduce the burden of biofilm-associated bacteria in the gallbladder, it may also lead to increased acute disease severity, sepsis, or even death. Fortunately, the data also demonstrated adverse outcomes are avoidable by pursuing a dual-therapy approach in which anti-biofilm compounds are administered in combination with traditional antibiotics. Future studies will focus on the identification of these compounds’ bacterial targets in order to understand their mechanisms of anti-biofilm activity and to develop compound analogs with enhanced attributes such as solubility or EC_50_/IC_50_. In addition, studies will be performed to determine if treatment cessation results in re-colonization of the gallbladder and if long-term carriage (e.g. >1 mo) can be similarly disrupted by the compounds and combination therapy.

## Materials & methods

### Ethics statement

Mouse care and housing was carried out in accordance with guidelines established by the Abigail Wexner Research Institute (AWRI) Institutional Animal Care and Use Committee (IACUC). All animal work was previously approved by AWRI IACUC (protocol AR18-00080). The AWRI IACUC is accredited by the Association for the Assessment and Accreditation of Laboratory Animal Care International (AAALAC). All research activities adhered to the statutes of the Animal Welfare Act and the guidelines of the Public Health Service as required in the *Guide for the Care and Use of Laboratory Animals*.

### Bacterial strains, growth conditions, and compounds

The *S*. Typhimurium strain ATCC 14028 (JSG210) and a RpoS^+^ *S*. Typhi strain (JSG4383, derived from the RpoS^-^ Ty2 strain; courtesy of Roy Curtiss III [52]) were streaked on Luria-Bertani (LB) agar plates and incubated at 37°C overnight. Individual bacterial colonies were used to inoculate LB or tryptic soy broth (TSB) for overnight (O/N) liquid cultures, which were grown at 37°C with aeration using a rotating drum.

Compound M4 was identified through a screen of 2-aminoimidazole derivatives and derivatives of other nitrogen-dense heterocycles as published previously [23]. Compound JG-1 was identified by screening a library of 4,000 small molecules (ChemBridge, San Diego, CA) in singlicate at a concentration of 5 μM for those that inhibited *S*. Typhimurium biofilm formation by ≥30% using the biofilm inhibition assay described below. The initial screen resulted in 226 hits, which were re-screened in triplicate to identify compounds that maintained ≥30% inhibitory activity. From this validation screen, JG-1 was identified as a lead compound for further study. Its synthesis is described in the supplemental information (S2 Fig).

Powdered JG-1 and M4 were stored dry and shielded from light. Stock solutions of these compounds were prepared in dimethyl sulfoxide (DMSO) to a concentration of 100 mM and stored at -20°C. Stock solutions were diluted into media or PBS as indicated such that the final concentration of DMSO was no greater than 5% (v/v). For experiments in which more than one concentration of anti-biofilm compound was assessed, the concentration of vehicle was standardized across all conditions by adding an appropriate volume of DMSO.

### Biofilm growth in microtiter plates and crystal violet staining

Biofilms were cultured using a modification of previously described procedures [53]. *S*. Typhimurium biofilms were grown on non-treated, flat-bottom polystyrene 96-well plates (Corning, Kennebunkport, ME) by normalizing O/N liquid cultures grown in LB to OD_600_ = 0.8 (~6.4 x 10^8^ CFU/mL), further diluting 1:100 into minimal media (TSB diluted 1:20 in ddH_2_O), and dispensing 100 μL/well. Plates were incubated at 30°C on a Fisherbrand™ nutating mixer (Thermo Fisher Scientific, Waltham, MA; 20° fixed angle, 24 rpm) for a total of 24 or 48 h as specified for each assay.

To simulate *S*. Typhi growth on gallstones, 96-well plates were pre-coated with cholesterol by adding 100 μL cholesterol solution (5 mg/mL, dissolved in equal proportions ethanol/isopropanol) per well and incubating overnight at room temperature to allow the solvent to evaporate. O/N liquid cultures of *S*. Typhi grown in TSB were normalized to OD_490_ = 0.65, further diluted 1:6 in TSB, and incubated statically for 3 h at 37°C. After the 3 h incubation, bacteria were diluted 1:2500 in TSB and added to cholesterol-coated 96-well plate (200μL/well). *S*. Typhi biofilms were incubated at 30°C on a nutating mixer (20° fixed angle, 24rpm) for 96 h as specified for each assay, with spent media removed and replaced with fresh media every 24 h.

Biofilm growth in microtiter plates was measured semi-quantitatively via crystal violet staining. After the designated incubation period, media and planktonic cells were discarded and microtiter plates were submerged in dH_2_O in order to wash away any remaining non-adherent bacteria. Biofilms were heat-fixed (1h, 60°C) prior to staining with a 33% crystal violet solution (6 mL PBS, 3.3 mL crystal violet, 333 μL methanol, 333 μL isopropanol) for 5 min. Plates were washed twice by submerging in dH_2_O, and the remaining bound crystal violet was solubilized with a solution of 33% glacial acetic acid. Biofilms were then quantified by measuring the optical density of the released dye at 570 nm using a spectrophotometer (Molecular Devices, SpectraMax M5).

### Biofilm inhibition and disruption assays

For inhibition assays, biofilms were grown as described above but with the designated concentration(s) of anti-biofilm compound (diluted in media from 100 mM stock solutions) or vehicle (DMSO) supplied in media either at the time of inoculation or at various timepoints 1–12 h post-inoculation. Biofilms were grown as described above for a total of 24 (*S*. Typhimurium) or 96 h (*S*. Typhi) prior to analysis via crystal violet staining or confocal microscopy.

For disruption assays, *S*. Typhi biofilms were initially grown in media alone for 24 (*S*. Typhimurium) or 96 h (*S*. Typhi) as described above. Spent media was then removed and replaced with media containing the designated concentration(s) of anti-biofilm compounds (diluted from 100 mM stock solutions) or vehicle (DMSO). The effects of ciprofloxacin addition on the biofilm-disrupting capabilities of JG-1 and M4 were also assessed by including additional treatment conditions in which vehicle or anti-biofilm compounds were combined with 0.25 μg/mL ciprofloxacin (the MIC for planktonic *S*. Typhimurium [31]). Biofilms were then incubated for an additional 24 h prior to analysis via crystal violet staining or confocal microscopy.

### IC_50_/EC_50_ determination

Half-maximal concentrations (the concentration of compound required to achieve a response halfway between baseline and maximum, defined here as 50% biofilm formed/remaining relative to the vehicle control for inhibition (IC_50_)/disruption (EC_50_), respectively) were calculated for both inhibition and disruption assays of *S*. Typhimurium and *S*. Typhi. For *S*. Typhimurium, IC_50_ values for biofilm inhibition were calculated using measurements of percent biofilm formed relative to vehicle (DMSO) after 24 h growth in the presence of various concentrations (2.5–100 μM) of JG-1 or M4 as described previously. IC_50_ values for inhibition of *S*. Typhi biofilms were calculated using measurements of percent biofilm formed relative to vehicle after a total of 96 h growth in the presence of various concentrations (3.1–200 μM) of JG-1 or M4 as described previously. EC_50_ values for disruption were calculated using measurements of percent biofilm remaining relative to vehicle after treating 24 h *S*. Typhimurium biofilms or 96 h *S*. Typhi biofilms (grown as described previously) with various concentrations (3.1–200 μM) of JG-1 or M4 for an additional 24h at 30°C. Biofilms were quantified using the crystal violet assay as described previously.

### Planktonic viability assay

To evaluate JG-1 and M4 for bactericidal or bacteriostatic capabilities, O/N liquid cultures of *S*. Typhimurium grown in LB were normalized to OD_600_ = 0.8 and diluted 1:1000 in LB containing JG-1, M4, or a vehicle control (DMSO). The effects of both low (10 μM) and high (100 μM) concentrations of JG-1 and M4 were assessed, and all conditions were normalized to a final DMSO concentration of 1% (v/v). Bacteria were incubated at 37°C with aeration, and samples taken over the course of 24 h were enumerated by serial dilution and plating onto LB agar (incubated for 16 h at 37°C).

### Confocal microscopy

In order to allow for optimal visualization of biofilms using confocal microscopy, *S*. Typhimurium biofilms were grown in 8-well chambered coverglasses (Thermo Fisher Scientific, Waltham, MA) by modifying the microtiter plate biofilm assay described previously. Briefly, *S*. Typhimurium O/N liquid cultures grown in LB were normalized and added into dilute media (TSB diluted 1:20 in ddH_2_O) as previously described and 200 μL of the bacterial dilution was added to coverglass wells. Chambered coverglasses were then incubated statically at 30°C for 96 h, with removal of spent media and addition of fresh dilute media every 24 h. After 96 h, spent media was removed and replaced with dilute media containing the specified concentration(s) of M4, JG-1, ciprofloxacin, M4 + ciprofloxacin, JG-1 + ciprofloxacin, or a vehicle control (DMSO), followed by an additional 24 h incubation at 30°C to allow for biofilm disruption.

Following the disruption period, media was removed and underlying biofilms were washed twice with Tris-buffered saline/Tween 20 (TBST). Bacterial cells and cellulose were labeled with the dyes Syto9 (final concentration 5 μM; Molecular Probes, Eugene, OR) and Calcofluor white (final concentration 30 μg/mL; Sigma-Aldrich, St. Louis, MO) supplied in TBST at the designated concentrations. After a 20 minute incubation period, the dye mixture was removed and biofilms were fixed with 2% paraformaldehyde solution for 30 minutes (Affimetrix, Cleveland, OH). Fixed biofilms were then blocked with 5% (w/v) bovine serum albumin (BSA) in TBST for 1 h at room temperature. For immunodetection of curli fimbriae, biofilms were incubated with human anti-amyloid IgG (diluted 1:200 in 5% BSA/TBST; courtesy of Çagla Tükel, Temple University) for 1 h, washed once with TBST, and incubated with Alexa Fluor 647 goat anti-human IgG (1:1000 in 5% BSA/TBST; Invitrogen, Waltham, MA) for 1 h. Secondary antibody was removed and replaced with TBST prior to imaging. All incubations were carried out at room temperature and samples were shielded from direct light to prevent photobleaching.

Stained biofilms were visualized at 100X magnification using an inverted Zeiss LSM 800 confocal laser scanning microscope. Three-dimensional biofilm structures were imaged by capturing three representative Z-stacks per well in three wells for each treatment. For every slice within a Z-stack, the signal for each fluorophore was recorded separately: Syto9-labeled *S*. Typhimurium were visualized at an excitation of 483 nm and an emission of 503 nm, Calcofluor white-bound cellulose was visualized at an excitation of 365 nm and an emission of 435 nm, and Alexa Fluor 647-labeled curli fimbriae were visualized at an excitation of 650 nm and an emission of 665 nm. Z-stacks were analyzed using Comstat2 software in order to calculate values of biomass, average thickness, and maximum thickness for each individual biofilm component assessed [32].

### Murine model of typhoid carriage and evaluation of the therapeutic efficacy anti-biofilm compounds -/+ ciprofloxacin

A total of 60 adult 129X1/SvJ NRAMP1^+/+^ mice (The Jackson Laboratory, Bar Harbour, ME) were used in this study. As described previously, mice were supplied a lithogenic diet (conventional mouse chow supplemented with 1% cholesterol and 0.5% cholic acid; Envigo, Indianapolis, IN) throughout the 8-wk period preceding infection in order to induce gallstone formation. Normalized liquid cultures of *S*. Typhimurium ATCC 14028 were serially diluted in sterile PBS to achieve an inoculum density of ~5×10^3^ CFU/mL, and mice were infected with ~10^3^ CFU by injecting 200 μL inoculum into the intraperitoneal (I.P.) cavity [6]. Exact bacterial density of the inoculum was determined via serial dilution and plating onto LB agar to allow for enumeration of CFUs after 16 h growth at 37°C.

Mice were randomly assigned to one of six groups corresponding to the following treatments: vehicle [5% (v/v) DMSO in PBS], 1 mg/kg/day ciprofloxacin, 10 mg/kg/day M4, 10 mg/kg/day JG-1, 10 mg/kg/day M4 + 1 mg/kg/day ciprofloxacin, or 10 mg/kg/day JG-1 + 1 mg/kg/day ciprofloxacin. Starting five days post-infection (dpi), daily treatments were administered parenterally into the I.P. cavity in a volume of 100 μL sterile PBS containing 5% (v/v) DMSO and continued for 10 days. On 15 dpi, mice were euthanized and their gallbladders, livers, and spleens were harvested and homogenized in a volume 500 μL sterile PBS using a TissueLyser LT bead mill (Qiagen, Valencia, CA). Tissue homogenates were serially diluted in sterile PBS, plated onto LB agar, and incubated at 37°C for 16 h, after which bacterial burdens were quantified by CFU enumeration. *Salmonella* fecal burden was also enumerated in order to investigate the effects of treatments on fecal shedding patterns. Feces were collected daily from 0 (immediately preceding infection) to 15 (immediately preceding euthanasia) dpi, homogenized in 1 mL sterile PBS using a TissueLyser LT bead mill (Qiagen), serially diluted in sterile PBS and plated onto xylose lysine deoxycholate (XLD) agar prior to incubation at 37°C for 16 h.

### Statistical information

For all *in vitro* assays, graphed data represents the average of at least three biological replicates, with error bars displaying the standard deviation. For mouse experiments, graphs were generated by combining the data from two separate experimental replicates; values for individual animals were plotted as data points and the corresponding group averages are graphed as lines. Statistical analyses of CFU enumeration experiments were conducted using log-transformed values. Data transformations and statistical analyses (described in figure legends) were performed using GraphPad Prism 8, and all p values < 0.05 were considered significant unless otherwise specified (i.e. when correcting for multiple comparisons).

## Supporting information

S1 Fig Differential enumeration of *Salmonella* within the gallbladder tissue and within the gallbladder contents of infected mice treated with JG-1 or M4. Mice were infected with *S*. Typhimurium as described previously in Fig 5 and administered a vehicle control (DMSO), 10mg/kg/day JG-1, or 10mg/kg/day M4 I.P. from 5–15 days post-infection (dpi). On 15 dpi, mice were euthanized and gallbladders were removed and lanced to release gallbladder contents (gallstones and bile), which was separated from gallbladder tissue prior to homogenization and plating onto LB agar for CFU enumeration. **A.** CFU enumeration of homogenized gallbladder tissue; **B.** CFU enumeration of homogenized gallbladder contents. ns non-significant, * p < 0.05, *** p < 0.001, **** p < 0.0001.
(TIF)

S2 Fig Supplemental Methods/Figures: Synthesis and Analysis of Lead Compound JG-1 General Information. All reactions were carried out under inert argon atmosphere with dry solvents unless otherwise noted. All commercial solvents and reagents were purchased from VWR, Sigma-Aldrich, Oakwood Chemical, or Matrix Scientific and used without further purification. Reactions were monitored by thin layer chromatography (TLC) using glass-backed pre-coated silica gel plates from VWR (TLC Silica Gel 60 Sheets, Millipore Sigma, F254, 60Å pore, 230–400 mesh) using UV visualization and ninhydrin stain as visualizing agent. Flash column chromatography was performed using silica gel (60Å, particle size 40–60 μm, VWR). Solvent system for compound purification was a mixture of ammonia-saturated methanol in methylene chloride with an initial methylene chloride column flush. Ammonia-saturated methanol was prepared by bubbling NH_3_ (Airgas) into methanol over the course of 15 minutes. Deuterated solvents for NMR characterization were purchased from Millipore Sigma via VWR. Deuterated chloroform was dried with molecular sieves from VWR (4Å, grade 514, mesh 8–12, Macron Fine Chemicals) before use and deuterated methanol was used as received. NMR spectra were recorded on a Bruker AVANCE III HD 400 Nanobay spectrometer or Bruker AVANCE III HD 500 without the use of signal suppression function and calibrated using the residual undeuterated solvent peak (CDCl_3_: δ 7.26 ppm ^1^H NMR, 77.16 ppm ^13^C NMR; CD_3_OD: δ 3.31 ppm ^1^H NMR, 49.00 ppm ^13^C NMR). Proton (^1^H) NMR is reported as follows: chemical shift in ppm (multiplicity [s = singlet, d = doublet, t = triplet, q = quartet, p = pentet, m = multiplet, br = broad], coupling constant(s) in Hz, relative integration). Carbon (^13^C) NMR data was reported as chemical shift (δ) in ppm. All NMR experiments were performed at ambient temperature. High resolution mass spectra (HRMS) were recorded on a Bruker micrOTOF II by electrospray ionization (ESI) time of flight experiments using direct infusion in 9:1 acetonitrile: water. Analysis was performed by the mass spectrometry and proteomics facility at University of Notre Dame and reported as *m/z*. **Part I: Addition of 1-Bromo-2-Fluorobenzene to Piperidine Core. *tert*-butyl ((1-(2-fluorophenethyl)piperidin-4-yl)methyl)(methyl)carbamate.** To a stirring solution of *tert*-butyl methyl(piperidin-4-ylmethyl)carbamate (100 mg, 0.438 mmol) in acetonitrile (ACN, 30 mL) under argon atmosphere was added oven-dried K_2_CO_3_ (182 mg, 1.31 mmol, 3 equiv) in one portion and the resulting mixture heated to reflux. 1-bromo-2-fluorobenzene (222 mg, 1.09 mmol, 2.5 equiv) was added neat in one portion and the reaction mixture was refluxed for 24 hours. The reaction was then cooled and methylene chloride (25 mL) was added. The mixture was evaporated *in vacuo* and redissolved in methylene chloride (50 mL). The resulting solution was extracted with deionized-H_2_O (3x30 mL) and then brine (30 mL). The organic layer was collected and dried over anhydrous sodium sulfate, filtered, evaporated *in vacuo*, and purified via flash column chromatography using 2% methanol saturated with ammonia in methylene chloride to obtain a yellow oil (132 mg, 0.376 mmol, 86%). ^1^H NMR (400 MHz, CDCl_3_) δ 7.184 (p, J = 7.7 Hz, 1H), δ 7.181 (p, J = 7.7 Hz, 1H), δ 7.05 (t, J = 9.0 Hz, 1H), δ 6.99 (t, J = 10.7 Hz, 1H), δ 3.10 (d, J = 6.9, 2H), δ 3.03 (d, br, J = 8.9, 2H) δ 2.88 (s, 5H), δ 2.59 (q, J = 5.3 Hz, 2H), δ 2.02 (t, br, J = 10.8, 2H), δ 1.64 (m, br, 3H), δ 1.44 (s, 9H), δ 1.33 (m, br, 2H). ^13^C NMR (400 MHz, CDCl_3_): 162.36, 159.93, 156.02, 131.03, 127.93, 127.85, 124.08, 115.37, 115.15, 79.38, 77.23, 59.07, 54.50, 53.44, 35.00, 34.72, 29.83, 28.48, 26.77. HRMS *m/z* calculated for C_20_H_31_FN_2_O_2_ [M+H]^+^: 351.24423, measured 351.24404. **Part II: Deprotection and Addition of Thiophene to 1. 1-(1-(2-fluorophenethyl)piperidin-4-yl)-*N*-methyl-*N*-(thiophen-3-ylmethyl)methanamine. 1** (132 mg, 0.376 mmol) was dissolved in 1 mL methylene chloride and 2 mL trifluoroacetic acid (TFA) was added. Reaction was allowed to stir under ambient temperature and atmosphere for 1 hour. Methanol (2 mL) was then added and mixture was again evaporated *in vacuo*. Addition of methanol was repeated four times until no further vapors were created upon addition of solvent and the vial contained a brown solid. Crude intermediate was dried for 18 hours under vacuum. The resulting solid was dissolved in 23 mL anhydrous ACN under argon, K_2_CO_3_ (207 mg, 1.50 mmol, 4 equiv) was added, and mixture was heated to reflux while stirring. 3-(bromomethyl)thiophene (79.7 mg, 0.45 mmol, 1.2 equiv) was added to 2 mL anhydrous ACN and was added to reaction dropwise over one hour. Reaction checked for completion by TLC after full addition of 3-(bromomethyl)thiophene, then cooled and methylene chloride (20 mL) was added. The mixture was evaporated *in vacuo* and redissolved in methylene chloride (50 mL). The resulting solution was extracted with deionized-H_2_O (3x30 mL) and the organic layer was washed with brine (30 mL), dried over anhydrous sodium sulfate, filtered, evaporated *in vacuo*, and purified via flash column chromatography using 2% methanol saturated with ammonia in methylene chloride to obtain a dark yellow oil (66 mg, 0.375 mmol, 51%). The pure product was then dissolved in methanol (1 mL) and glacial hydrochloric acid (0.1 mL) was added to make a salt. The product was evaporated *in vacuo*. Methanol addition and subsequent evaporation was repeated six times until a yellow solid was obtained and product was dried under vacuum for 24 hours to yield the salt of the pure product. ^1^H NMR (500 MHz, CD_3_OD) δ 7.35 (dd, J = 4.9, 3.0 Hz, 1H), δ 7.26 (dt, J = 7.6, 1.7 Hz, 1H), δ 7.23–7.20 (m, 2H) δ 7.09 (dt, J = 11.2, 1.2 Hz, 1H), δ 7.07 (dd, J = 4.9, 1.2 Hz, 1H), δ 7.04 (qd, J = 9.6, 1.2 Hz, 1H), δ 3.54 (s, 2H), δ 3.03 (d, br, J = 11.6, 2H), δ 2.88–2.83 (m, 2H), δ 2.60–2.57 (m, 2H), δ 2.23 (s, 1H), δ 2.22 (s, 3H), δ 2.12 (t, br, J = 11.5, 2H), δ 1.83 (d, br, J = 12.2, 2H), δ 1.61 (m, J = 3.7, 1H), δ 1.21 (qd, J = 24.9, 3.7, 2H). ^13^C NMR (500 MHz, CD_3_OD): 132.24, 132.21, 131.02, 130.74, 130.68, 130.18, 128.98, 125.98, 116.69, 116.52, 60.80, 57.69, 55.63, 53.15, 53.06, 41.08, 30.66, 28.57, 28.49, 25.02, 25.00. HRMS *m/z* calculated for C_20_H_27_FN_2_S [M+H]^+^: 347.1952, measured 347.1945.
(DOCX)
