# Supplementary figures and images for: A dual-therapy approach for the treatment of biofilm-mediated Salmonella gallbladder carriage
Source: PLoS Pathog. 2020 Dec 28;16(12):e1009192. doi: 10.1371/journal.ppat.1009192 (PMC7793255; doi:10.1371/journal.ppat.1009192)

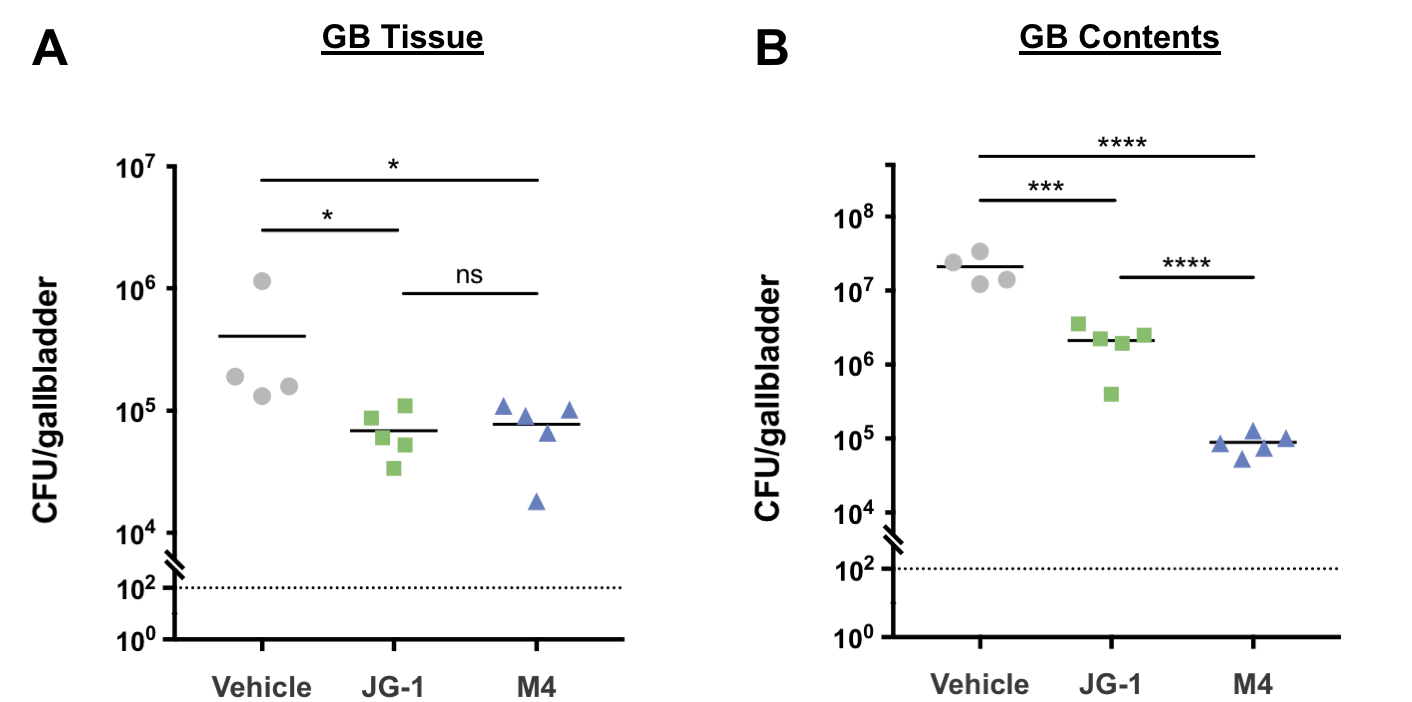

Supplement: S1 Fig — Mice were infected with S. Typhimurium as described previously in Fig 5 and administered a vehicle control (DMSO), 10mg/kg/day JG-1, or 10mg/kg/day M4 I.P. from 5–15 days post-infection (dpi). On 15 dpi, mice were euthanized and gallbladders were removed and lanced to release gallbladder contents (gallstones and bile), which was separated from gallbladder tissue prior to homogenization and plating onto LB agar for CFU enumeration. A. CFU enumeration of homogenized gallbladder tissue; B. CFU enumeration of homogenized gallbladder contents. ns non-significant, * p < 0.05, *** p < 0.001, **** p < 0.0001. (TIF) [file ppat.1009192.s001.tif]
