# Supplementary material for: A dual-therapy approach for the treatment of biofilm-mediated Salmonella gallbladder carriage
Source: PLoS Pathog. 2020 Dec 28;16(12):e1009192. doi: 10.1371/journal.ppat.1009192 (PMC7793255; doi:10.1371/journal.ppat.1009192)
Supplement: S2 Fig — All reactions were carried out under inert argon atmosphere with dry solvents unless otherwise noted. All commercial solvents and reagents were purchased from VWR, Sigma-Aldrich, Oakwood Chemical, or Matrix Scientific and used without further purification. Reactions were monitored by thin layer chromatography (TLC) using glass-backed pre-coated silica gel plates from VWR (TLC Silica Gel 60 Sheets, Millipore Sigma, F254, 60Å pore, 230–400 mesh) using UV visualization and ninhydrin stain as visualizing agent. Flash column chromatography was performed using silica gel (60Å, particle size 40–60 μm, VWR). Solvent system for compound purification was a mixture of ammonia-saturated methanol in methylene chloride with an initial methylene chloride column flush. Ammonia-saturated methanol was prepared by bubbling NH3 (Airgas) into methanol over the course of 15 minutes. Deuterated solvents for NMR characterization were purchased from Millipore Sigma via VWR. Deuterated chloroform was dried with molecular sieves from VWR (4Å, grade 514, mesh 8–12, Macron Fine Chemicals) before use and deuterated methanol was used as received. NMR spectra were recorded on a Bruker AVANCE III HD 400 Nanobay spectrometer or Bruker AVANCE III HD 500 without the use of signal suppression function and calibrated using the residual undeuterated solvent peak (CDCl3: δ 7.26 ppm 1H NMR, 77.16 ppm 13C NMR; CD3OD: δ 3.31 ppm 1H NMR, 49.00 ppm 13C NMR). Proton (1H) NMR is reported as follows: chemical shift in ppm (multiplicity [s = singlet, d = doublet, t = triplet, q = quartet, p = pentet, m = multiplet, br = broad], coupling constant(s) in Hz, relative integration). Carbon (13C) NMR data was reported as chemical shift (δ) in ppm. All NMR experiments were performed at ambient temperature. High resolution mass spectra (HRMS) were recorded on a Bruker micrOTOF II by electrospray ionization (ESI) time of flight experiments using direct infusion in 9:1 acetonitrile: water. Analysis was performed by the mass [file ppat.1009192.s002.docx]

**S2 Fig. Supplemental Methods/Figures: Synthesis and Analysis of Lead Compound JG-1**

**General Information**

All reactions were carried out under inert argon atmosphere with dry solvents unless otherwise noted. All commercial solvents and reagents were purchased from VWR, Sigma-Aldrich, Oakwood Chemical, or Matrix Scientific and used without further purification. Reactions were monitored by thin layer chromatography (TLC) using glass-backed pre-coated silica gel plates from VWR (TLC Silica Gel 60 Sheets, Millipore Sigma, F254, 60Å pore, 230-400 mesh) using UV visualization and ninhydrin stain as visualizing agent. Flash column chromatography was performed using silica gel (60Å, particle size 40-60 μm, VWR). Solvent system for compound purification was a mixture of ammonia-saturated methanol in methylene chloride with an initial methylene chloride column flush. Ammonia-saturated methanol was prepared by bubbling NH_3_ (Airgas) into methanol over the course of 15 minutes. Deuterated solvents for NMR characterization were purchased from Millipore Sigma via VWR. Deuterated chloroform was dried with molecular sieves from VWR (4Å, grade 514, mesh 8-12, Macron Fine Chemicals) before use and deuterated methanol was used as received. NMR spectra were recorded on a Bruker AVANCE III HD 400 Nanobay spectrometer or Bruker AVANCE III HD 500 without the use of signal suppression function and calibrated using the residual undeuterated solvent peak (CDCl_3_: δ 7.26 ppm ^1^H NMR, 77.16 ppm ^13^C NMR; CD_3_OD: δ 3.31 ppm ^1^H NMR, 49.00 ppm ^13^C NMR). Proton (^1^H) NMR is reported as follows: chemical shift in ppm (multiplicity [s = singlet, d = doublet, t = triplet, q = quartet, p = pentet, m = multiplet, br = broad], coupling constant(s) in Hz, relative integration). Carbon (^13^C) NMR data was reported as chemical shift (δ) in ppm. All NMR experiments were performed at ambient temperature. High resolution mass spectra (HRMS) were recorded on a Bruker micrOTOF II by electrospray ionization (ESI) time of flight experiments using direct infusion in 9:1 acetonitrile: water. Analysis was performed by the mass spectrometry and proteomics facility at University of Notre Dame and reported as *m/z*.

**Part I: Addition of 1-Bromo-2-Fluorobenzene to Piperidine Core**


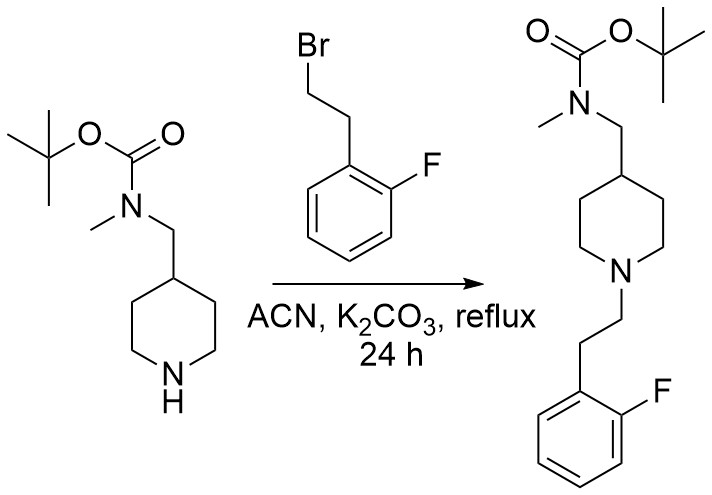


**(1)**

***tert*-butyl ((1-(2-fluorophenethyl)piperidin-4-yl)methyl)(methyl)carbamate.** To a stirring solution of *tert*-butyl methyl(piperidin-4-ylmethyl)carbamate (100 mg, 0.438 mmol) in acetonitrile (ACN, 30 mL) under argon atmosphere was added oven-dried K_2_CO_3_ (182 mg, 1.31 mmol, 3 equiv) in one portion and the resulting mixture heated to reflux. 1-bromo-2-fluorobenzene (222 mg, 1.09 mmol, 2.5 equiv) was added neat in one portion and the reaction mixture was refluxed for 24 hours. The reaction was then cooled and methylene chloride (25 mL) was added. The mixture was evaporated *in vacuo* and redissolved in methylene chloride (50 mL). The resulting solution was extracted with deionized-H_2_O (3x30 mL) and then brine (30 mL). The organic layer was collected and dried over anhydrous sodium sulfate, filtered, evaporated *in vacuo*, and purified via flash column chromatography using 2% methanol saturated with ammonia in methylene chloride to obtain a yellow oil (132 mg, 0.376 mmol, 86%). ^1^H NMR (400 MHz, CDCl_3_) δ 7.184 (p, J = 7.7 Hz, 1H), δ 7.181 (p, J = 7.7 Hz, 1H), δ 7.05 (t, J = 9.0 Hz, 1H), δ 6.99 (t, J = 10.7 Hz, 1H), δ 3.10 (d, J = 6.9, 2H), δ 3.03 (d, br, J = 8.9, 2H) δ 2.88 (s, 5H), δ 2.59 (q, J = 5.3 Hz, 2H), δ 2.02 (t, br, J = 10.8, 2H), δ 1.64 (m, br, 3H), δ 1.44 (s, 9H), δ 1.33 (m, br, 2H). ^13^C NMR (400 MHz, CDCl_3_): 162.36, 159.93, 156.02, 131.03, 127.93, 127.85, 124.08, 115.37, 115.15, 79.38, 77.23, 59.07, 54.50, 53.44, 35.00, 34.72, 29.83, 28.48, 26.77. HRMS *m/z* calculated for C_20_H_31_FN_2_O_2_ [M+H]^+^: 351.24423, measured 351.24404.

**Part II: Deprotection and Addition of Thiophene to 1**

2 HCl

**(2)**

**(1)**


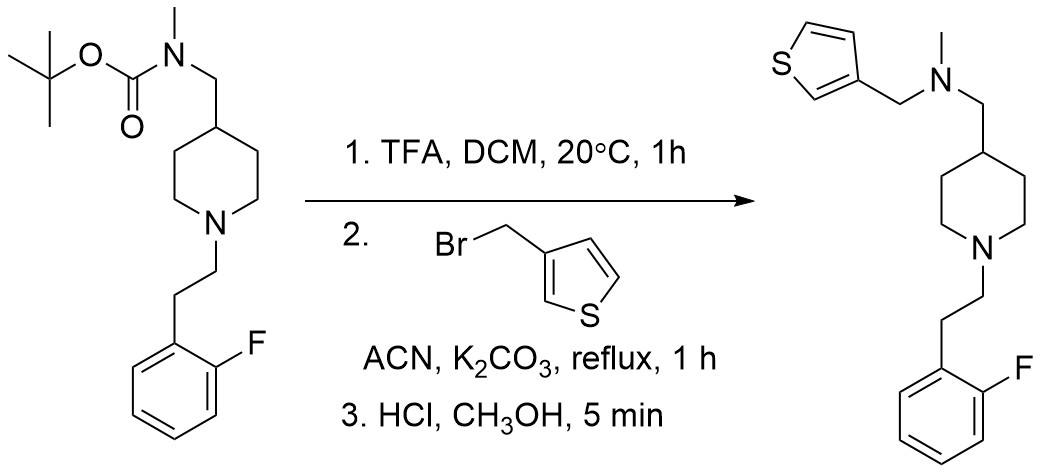


**1-(1-(2-fluorophenethyl)piperidin-4-yl)-*N*-methyl-*N*-(thiophen-3-ylmethyl)methanamine.** **1** (132 mg, 0.376 mmol) was dissolved in 1 mL methylene chloride and 2 mL trifluoroacetic acid (TFA) was added. Reaction was allowed to stir under ambient temperature and atmosphere for 1 hour. Methanol (2 mL) was then added and mixture was again evaporated *in vacuo*. Addition of methanol was repeated four times until no further vapors were created upon addition of solvent and the vial contained a brown solid. Crude intermediate was dried for 18 hours under vacuum. The resulting solid was dissolved in 23 mL anhydrous ACN under argon, K_2_CO_3_ (207 mg, 1.50 mmol, 4 equiv) was added, and mixture was heated to reflux while stirring. 3-(bromomethyl)thiophene (79.7 mg, 0.45 mmol, 1.2 equiv) was added to 2 mL anhydrous ACN and was added to reaction dropwise over one hour. Reaction checked for completion by TLC after full addition of 3-(bromomethyl)thiophene, then cooled and methylene chloride (20 mL) was added. The mixture was evaporated *in vacuo* and redissolved in methylene chloride (50 mL). The resulting solution was extracted with deionized-H_2_O (3x30 mL) and the organic layer was washed with brine (30 mL), dried over anhydrous sodium sulfate, filtered, evaporated *in vacuo*, and purified via flash column chromatography using 2% methanol saturated with ammonia in methylene chloride to obtain a dark yellow oil (66 mg, 0.375 mmol, 51%). The pure product was then dissolved in methanol (1 mL) and glacial hydrochloric acid (0.1 mL) was added to make a salt. The product was evaporated *in vacuo*. Methanol addition and subsequent evaporation was repeated six times until a yellow solid was obtained and product was dried under vacuum for 24 hours to yield the salt of the pure product. ^1^H NMR (500 MHz, CD_3_OD) δ 7.35 (dd, J = 4.9, 3.0 Hz, 1H), δ 7.26 (dt, J = 7.6, 1.7 Hz, 1H), δ 7.23-7.20 (m, 2H) δ 7.09 (dt, J = 11.2, 1.2 Hz, 1H), δ 7.07 (dd, J = 4.9, 1.2 Hz, 1H), δ 7.04 (qd, J = 9.6, 1.2 Hz, 1H), δ 3.54 (s, 2H), δ 3.03 (d, br, J = 11.6, 2H), δ 2.88-2.83 (m, 2H), δ 2.60-2.57 (m, 2H), δ 2.23 (s, 1H), δ 2.22 (s, 3H), δ 2.12 (t, br, J = 11.5, 2H), δ 1.83 (d, br, J = 12.2, 2H), δ 1.61 (m, J = 3.7, 1H), δ 1.21 (qd, J = 24.9, 3.7, 2H). ^13^C NMR (500 MHz, CD_3_OD): 132.24, 132.21, 131.02, 130.74, 130.68, 130.18, 128.98, 125.98, 116.69, 116.52, 60.80, 57.69, 55.63, 53.15, 53.06, 41.08, 30.66, 28.57, 28.49, 25.02, 25.00. HRMS *m/z* calculated for C_20_H_27_FN_2_S [M+H]^+^: 347.1952, measured 347.1945.


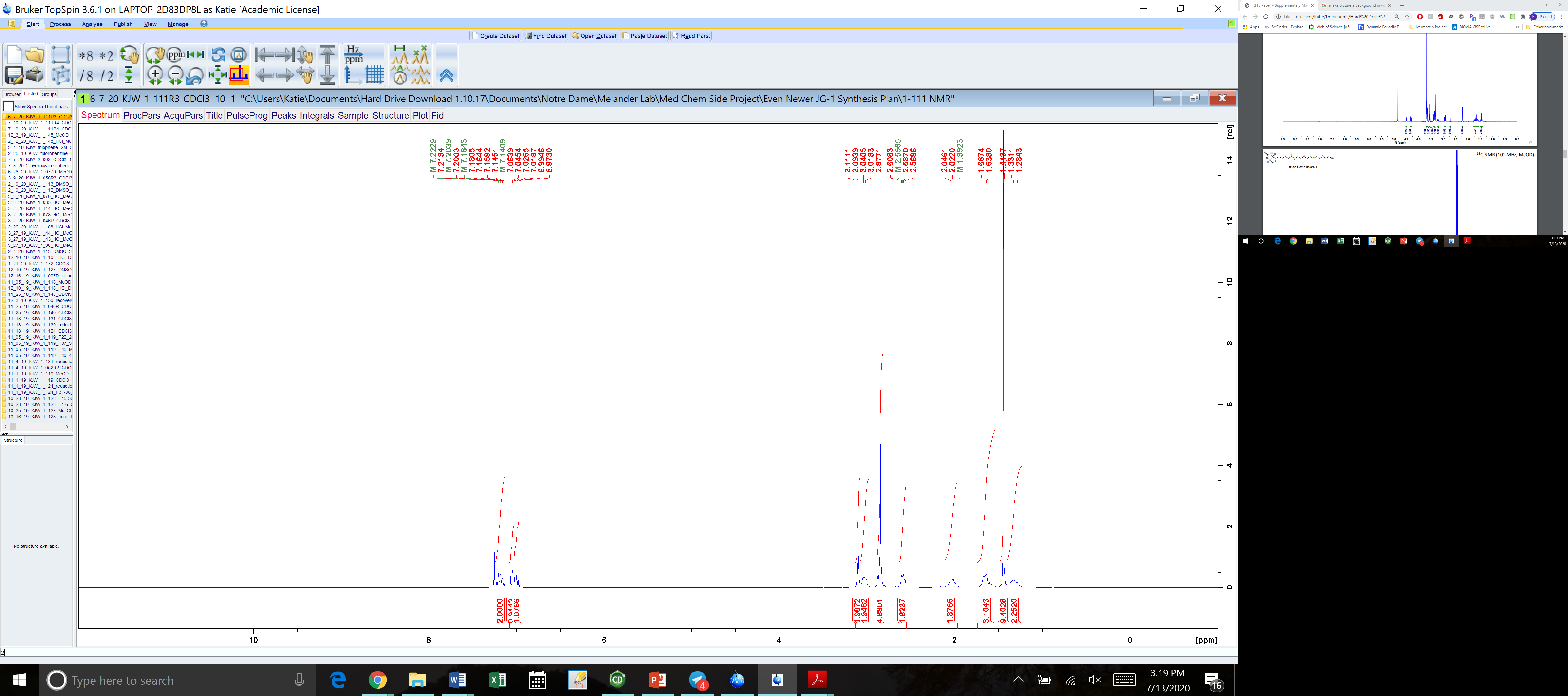
^1^H NMR (400 MHz, CDCl_3_)


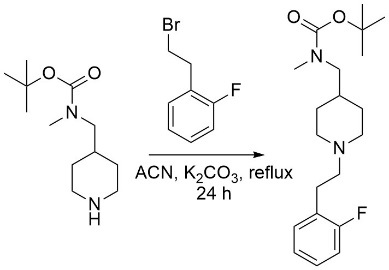


**1**

^13^C NMR (400 MHz, CDCl_3_)

**1**


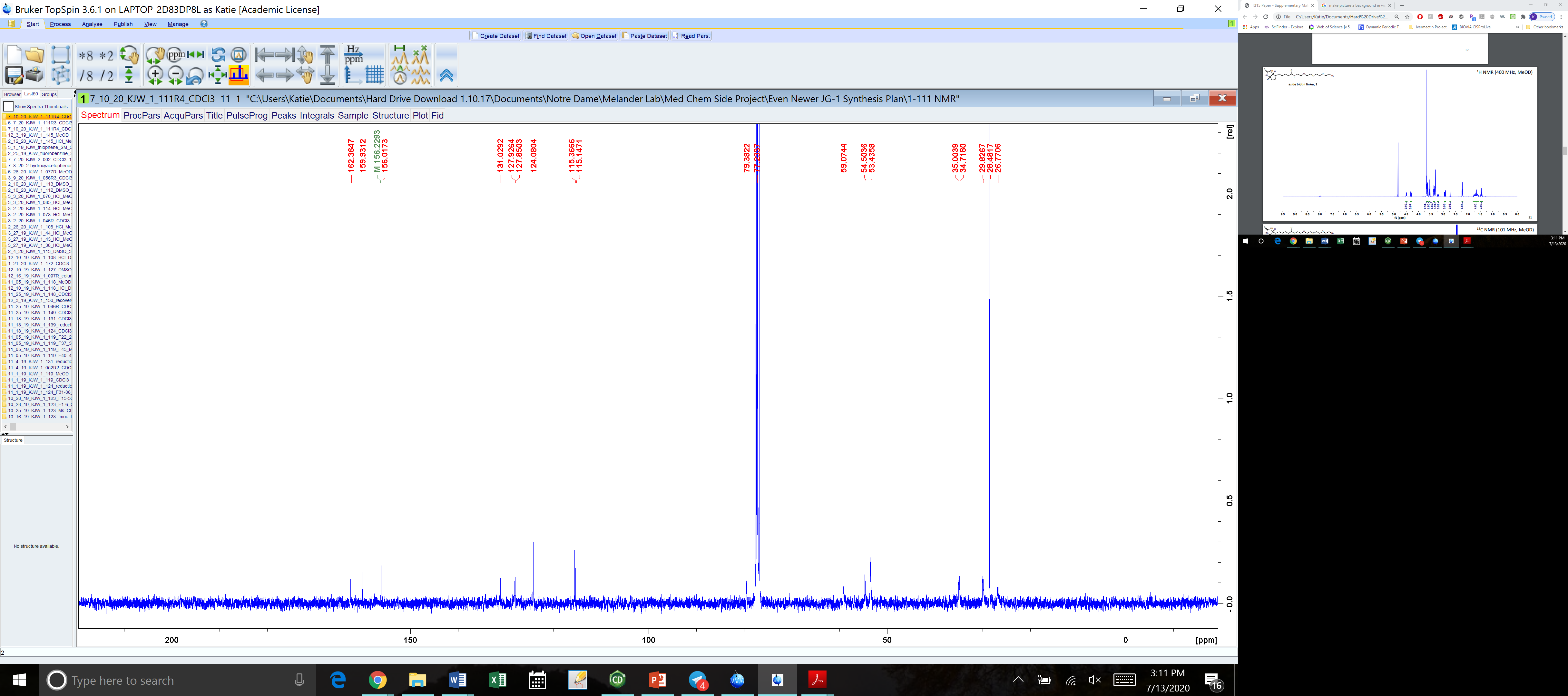

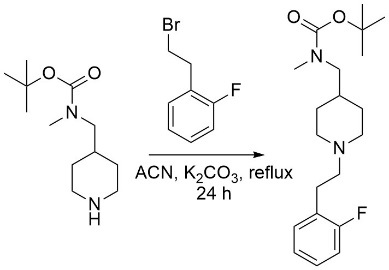


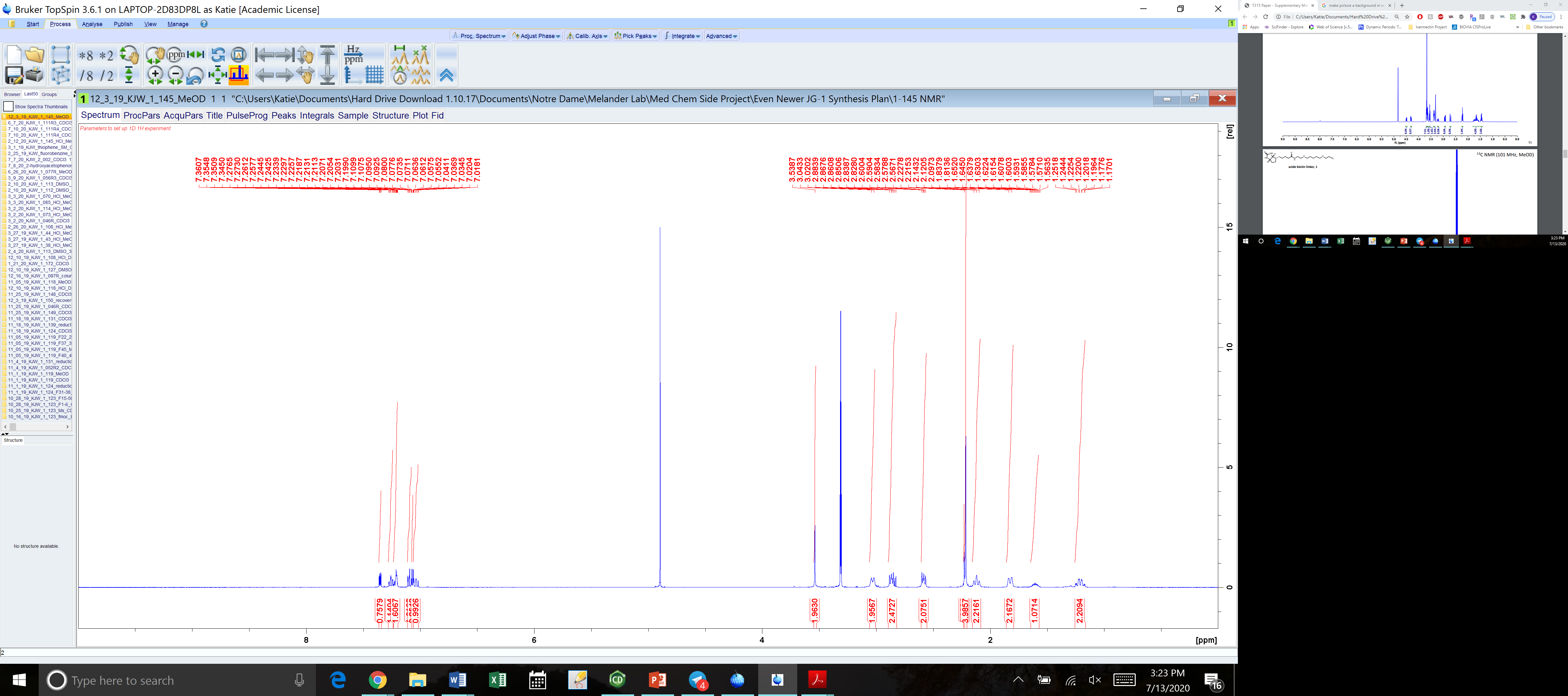
^1^H NMR (500 MHz, CD_3_OD)


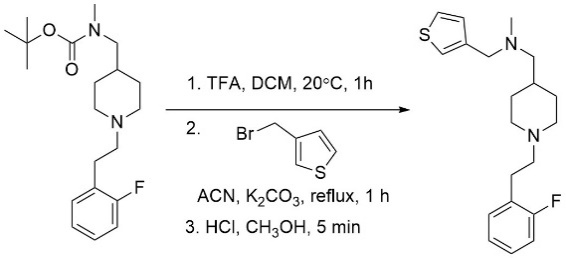


**2**


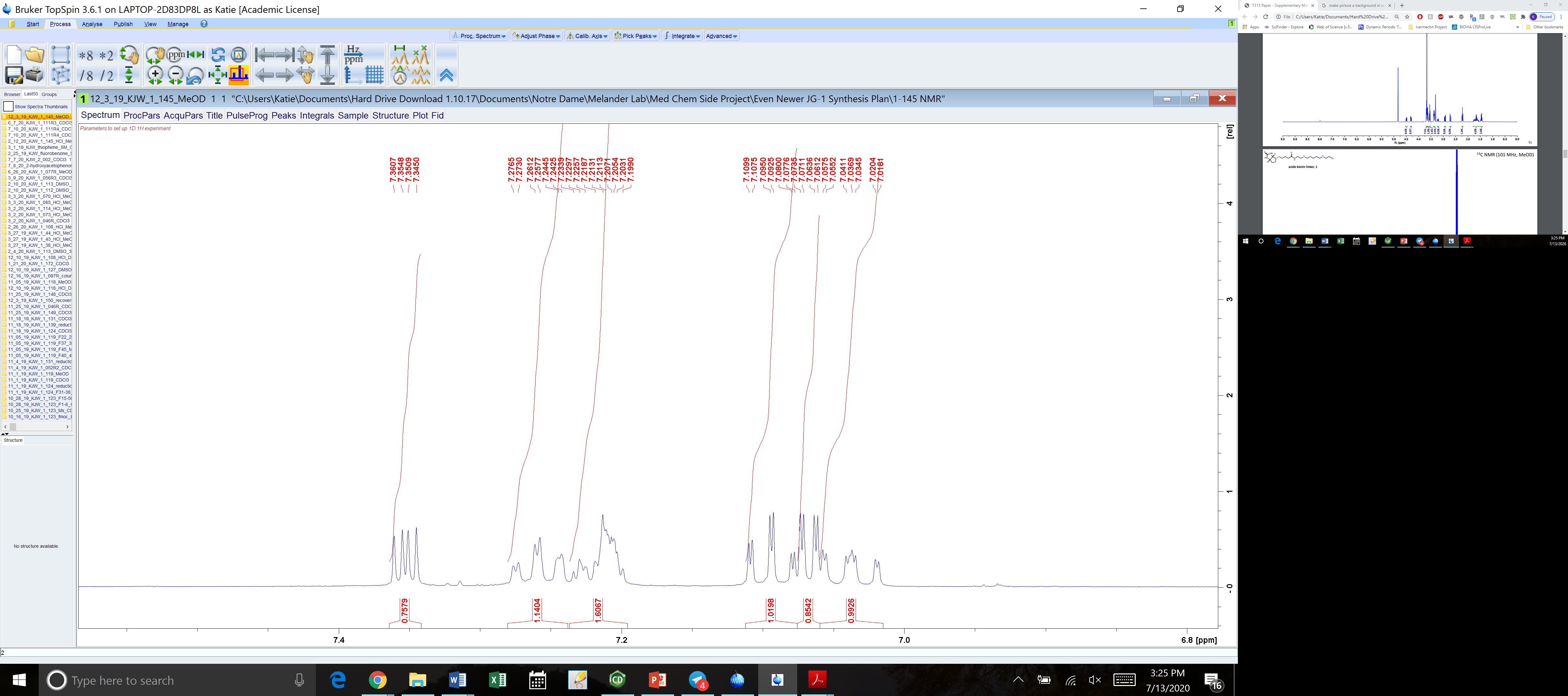
^1^H NMR (500 MHz, CD_3_OD), detail


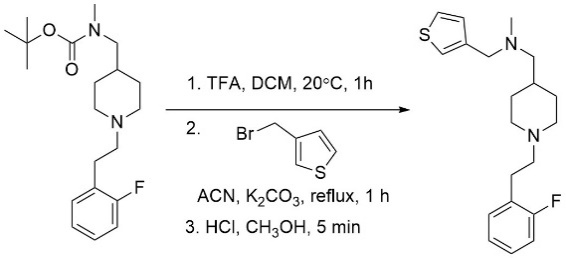


**2**


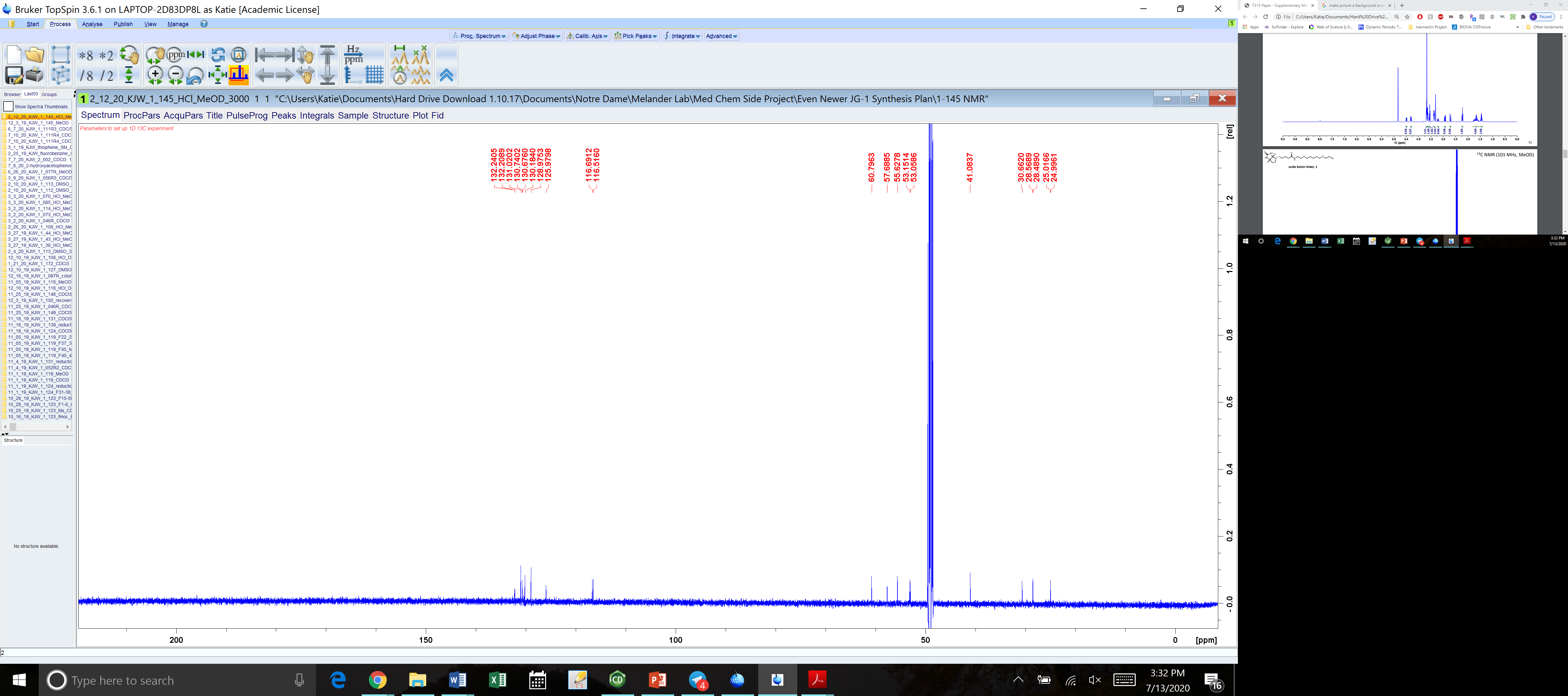
^13^C NMR (500 MHz, CD_3_OD)


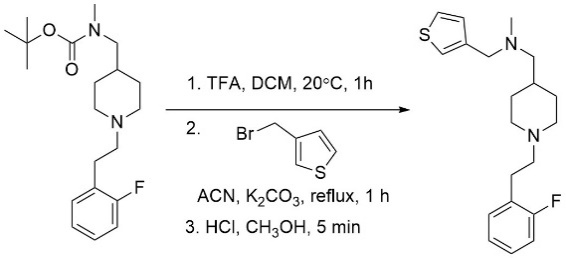


**2**
